# Sensing Levofloxacin with an RNA Aptamer as a Bioreceptor

**DOI:** 10.3390/bios14010056

**Published:** 2024-01-22

**Authors:** Janice Kramat, Leon Kraus, Vincent J. Gunawan, Elias Smyej, Philipp Froehlich, Tim E. Weber, Dieter Spiehl, Heinz Koeppl, Andreas Blaeser, Beatrix Suess

**Affiliations:** 1 Synthetic RNA Biology, Department of Biology, Technical University of Darmstadt, 64287 Darmstadt, Germany; 2 Self-Organizing Systems, Department of Electrical Engineering and Information Technology, Technical University of Darmstadt, 64283 Darmstadt, Germany; 3 Institute for BioMedical Printing Technologies, Technical University of Darmstadt, 64289 Darmstadt, Germany; 4 Centre for Synthetic Biology, Technical University of Darmstadt, 64289 Darmstadt, Germany

**Keywords:** aptamers, SELEX, antibiotics, levofloxacin, lateral flow assay, biosensor

## Abstract

To combat the growing threat of antibiotic resistance, environmental testing for antibiotic contamination is gaining an increasing role. This study aims to develop an easy-to-use assay for the detection of the fluoroquinolone antibiotic levofloxacin. Levofloxacin is used in human and veterinary medicine and has been detected in wastewater and river water. An RNA aptamer against levofloxacin was selected using RNA Capture-SELEX. The 73 nt long aptamer folds into three stems with a central three-way junction. It binds levofloxacin with a *K_d_* of 6 µM and discriminates the closely related compound ciprofloxacin. Furthermore, the selection process was analyzed using a next-generation sequencing approach to better understand the sequence evolution throughout the selection. The aptamer was used as a bioreceptor for the development of a lateral flow assay. The biosensor exploited the innate characteristic of RNA Capture-SELEX to select aptamers that displace a complementary DNA oligonucleotide upon ligand binding. The lateral flow assay achieved a limit of visual detection of 100 µM. While the sensitivity of this assay constrains its immediate use in environmental testing, the present study can serve as a template for the selection of RNA aptamer-based biosensors.

## 1. Introduction

Antimicrobial resistance has become a global issue that increasingly poses significant dangers to public health. Infections by resistant bacteria such as vancomycin-resistant *Enterococcus* (VRE) and methicillin-resistant *Staphylococcus aureus* (MRSA) do not only entail cost-intensive treatment but often end in the death of the infected [1,2]. The emergence and spread of antibiotic resistance are complex processes influenced by various factors. Major contributors are the over-prescription of antibiotics in human medicine and their use in animal livestock [3,4]. Furthermore, the use of antibiotics in agriculture and aquaculture has been linked to the dissemination of antibiotic-resistant bacteria [5]. This has already led to millions of deaths each year [2]. As these numbers are only projected to rise, it is paramount to stop the spread of new antibiotic resistance mechanisms. One component in achieving this is the reduction in antibiotic overuse. Another factor is environmental testing for the antibiotic burden of soil and water for monitoring the development of new antibiotic resistance mechanisms from ever evolving. The main methods for detecting antibiotics and their metabolites in the environment have been cumbersome and expensive laboratory analyses using MS and LC-MS [5]. While recent advances in biosensing have led to numerous excellent antibiotic sensors, many of them lack the modularity to detect the diversity of compounds and the ease of use needed for their use by minimally trained personnel in the field [6]. This study aims to provide a protocol for the straightforward development of RNA aptamer-based lateral flow assays (LFAs) for the detection of antibiotics.

RNA aptamers are a fairly modern part of the field of biosensing [7]. These single-stranded oligonucleotides can selectively bind different types of ligands with high affinity, which makes them an excellent choice for use as bioreceptors in sensing devices [8,9]. Aptamers can, in principle, be selected to bind any target and, once selected, they can easily be manufactured in vitro with low batch-to-batch variability. They have been used to detect pathogens [10], proteins [11], drugs [12], antibiotics [13], and other analytes [14].

An effective method to generate aptamers is the Systematic Evolution of Ligands by Exponential Enrichment (SELEX) [15,16]. This process was originally published in the early nineties and has since developed into numerous protocols for many different applications [17]. One very suitable method for the selection of biosensing aptamers is called Capture-SELEX [18,19,20,21,22]. Here, the RNA library is immobilized via a complementary DNA capture oligonucleotide (Capture-ON). Upon ligand binding to the aptamer, this oligonucleotide is displaced, and the aptamer is released. This offers two key benefits for the selection of aptamers intended for biosensing. Firstly, this method does not rely on immobilized target compounds, meaning that the ligands are used in solution and do not need to be chemically altered for the selection. This is highly beneficial for the selection of aptamers that bind small molecules, such as antibiotics. Secondly, aptamers generated in this way have to undergo an innate structural change upon ligand binding. This is a direct consequence of the selection method using a Capture-ON: only RNA sequences that undergo structural refolding can displace the oligonucleotide and are released in the SELEX process.

As a proof of concept, this study focused on the fluoroquinolone antibiotic levofloxacin (LFX) as the target molecule for an RNA aptamer. LFX is used in both human and veterinary medicine. It has been on the WHO’s list of essential medicines since 2019 [23]. Due to its pronounced effect against Gram-positive bacteria and certain pathogens, it is used to treat pneumonia, chronic bronchitis, skin inflammations, urinary tract infections, and other maladies [24]. LFX is also gaining interest in veterinary medicine, a common breeding ground for antibiotic resistance mechanisms [25,26,27]. It has been detected in wastewater, but also in river water [28] where it can severely affect aquatic microbial life and may promote antibiotic resistance in aquatic bacteria [29]. Given these emerging issues, a simple-to-use assay for the detection of LFX could aid in the prevention of widespread resistance against LFX.

RNA Capture-SELEX selects aptamers that have the intrinsic capability to hybridize with the Capture-ON but detach from it when binding their respective ligands. Hybridized aptamer and Capture-ON can be held in solution and free Capture-ON is detectable after analyte addition. This principle can be used as an effective indicator for the presence of an analyte, in this case, LFX. Based on the work of Ying and colleagues an LFA was developed to detect free Capture-ON and thereby the presence of LFX [30]. LFAs offer simple and cost-effective detection of water contaminants. They are portable, do not require laboratory equipment, and can be used by untrained personnel, which makes them uniquely suited for environmental testing [31,32]. The LFX-sensing LFA uses gold nanoparticles for a visual readout and Hybridization Chain Reaction (HCR) for signal amplification [30,33].

We herein demonstrate the selection and engineering of a novel LFX binding aptamer and its use as a bioreceptor in an easy-to-use LFA.

## 2. Materials and Methods

### 2.1. Pool Preparation and In Vitro Selection Using Capture-SELEX

The DNA pool used for the selection contained a 13-nucleotide (nt)-long constant region (docking sequence) flanked by two randomized regions of 40 nt (5’) and 10 nt (3’). Two constant regions needed for PCR amplification complemented the pool, with 27 nucleotides (nt) at the 5’ end and 33 nt at the 3’ end. The RNA pool therefore had the following sequence: 5′-GGGCACUCCAAGCUAGAUCUACCGGU-N40-CUACUGGCUUCUA-N10-AAAAUGGCUAGCAAAGGAGAAGAACUUUUCACU-3′. The pool preparation involved the amplification of a DNA template pool (purchased from Sigma-Aldrich, St. Louis, MO, USA; PAGE purified) via PCR and subsequent in vitro transcription to produce the required RNA pool.

The in vitro selection was performed using the Capture-SELEX method, for a detailed protocol see [19,20,34]. The RNA pool was radioactively labeled at the 5′-end using γ32P-ATP to measure elution fractions during the selection process. RNA molecules of 1 × 10^15^ were hybridized to a biotinylated DNA Capture-ON complementary to the docking sequence. The hybrids were then immobilized on streptavidin-coated magnetic beads. The subsequent selection included three washing steps with Capture-SELEX Buffer (5x CSB: 200 mM HEPES, 1.25 mM KCl, 100 mM NaCl, and 25 mM MgCl_2_, adjusted to pH 7.4 with HCl) and, at different stages of the SELEX, elution with 0.1/1 mM LFX and counter elution with 0.1 mM of ciprofloxacin (CFX). For the amplification of the eluted RNA molecules, reverse transcription PCR (RT-PCR) was performed. At the end of each selection round, in vitro transcription was carried out using the obtained PCR product as a DNA template. The resulting RNA pool was used to start a new round of SELEX. See the Supplementary Materials, Figure S1, for an illustration of the RNA Capture-SELEX process.

### 2.2. Next-Generation Sequencing

For deep sequencing of the entire selection process using next-generation sequencing (NGS), the individual rounds of SELEX were prepared as follows. For amplification of double-stranded DNA (dsDNA), 8 µL of the RT-PCR product of each individual round was mixed with 20 µL of 5x Q5^®^ reaction buffer (NEB), 200 µM of dNTPs, 0.5 µM of oligonucleotide pool_fwd (5’-CCAAGTAATACGACTCACTATAGGGCAACTCCAAGCTAGATCTACCGGT-3’), 0.5 µM of oligonucleotide pool_rev (5’-AGTGAAAAGTTCTCCTTTGCTAGCCATTTT-3’), and 2 U of Q5^®^ high-fidelity DNA polymerase (NEB) and ultrapure water (filled up to 100 µL). After performing PCR (30 s at 98 °C, 5 cycles of (10 s at 98 °C, 20 s at 55 °C, and 20 s at 72 °C), and 2 min at 72 °C), gel electrophoresis was performed to exclude overamplification. Subsequently, the samples were purified using Promega’s Wizard^®^ SV Gel and the PCR Clean-Up system. The concentrations were determined via spectrophotometric measurement using a NanoPhotometer^®^ (IMPLEN, Munich, Germany) and adjusted to 20 ng/µL with ultrapure water. Illumina sequencing was performed by the company GENEWIZ^®^ (Azenta Life Sciences, Burlington, MA, USA), using their Amplicon-EZ service.

A total of 1.5 million sequences were obtained of which 1.3 million remained after the filtering steps of preprocessing, consisting of 21 rounds of SELEX plus one native pool. Preprocessing was conducted by first merging paired-end reads using the mergePairs function, a feature grounded in the work of Edgar and Flyvbjerg [35], and implemented in the vsearch tool developed by Rognes et al. [36]. This function integrates quality controls, such as constraints on maximum expected errors, and merges length limits. Following this, the sequences were oriented according to a specific template using the OrientNucleotides function. This function is part of the DECIPHER package in R [37]. The final preprocessing step effectively reduced data noise by filtering out approximately 4% of sequences that significantly deviated from the template (distance of more than 10).

Sequence frequencies (i.e., the reads in the corresponding rounds) within each round were established, with normalization against total read count providing a measure of reads per million (RPM). Additionally, for each sequence identified as a top sequence in the final round, its frequency (in RPM) in every round was tracked. The calculation of cumulative distribution functions (CDFs) from normalized sequence frequencies enabled the generation of Lorenz curves, providing a visual representation of sequence abundance distribution in the selection pool. Both the CDF of RPM and the rank in the library were scaled to fall between zero and one. A diagram with the bioinformatic workflow can be found in the Supplementary Materials, Figure S2, and a boxplot of the effective error distributions is shown in the Supplementary Materials, Figure S3. Supplementary Tables S1–S3 contain the relevant statistics.

### 2.3. RNA Synthesis for In Vitro Analysis

The production of RNA to be used for elution assays and isothermal titration calorimetry (ITC) measurements were carried out using in vitro transcription on varying scales. The DNA templates were ordered as single-stranded DNA (ssDNA) oligonucleotides from Sigma-Aldrich. Respective ssDNA oligonucleotides were combined with equal molarity, incubated at 95 °C for 5 min, and allowed to cool slowly to room temperature. The resulting dsDNA templates contained a T7 promoter followed by the specific sequence for the respective aptamer candidate. In vitro transcription was performed by mixing a dsDNA template (3 µg/mL) with 20 mM of Mg(Ac)_2_, 200 mM of Tris-HCl pH 8.0, 20 mM of DTT, 2 mM of spermidine, 4 mM of each NTP, 15% of DMSO, and 1000 U/mL of T7 polymerase (self-made) and ultrapure water (filled up to the final volume). After incubation for 6 h at 37 °C, the RNA was purified via excision from a polyacrylamide gel. The resulting RNA concentration was determined using spectrophotometric measurement with a NanoPhotometer^®^ (IMPLEN).

### 2.4. Isothermal Titration Calorimetry

For ITC measurements, the same buffer composition was used as for the Capture-SELEX method. The measurements were carried out with 500 µM of LFX and 30 µM of RNA. Refolding of the aptamers was always carried out before the measurement: RNA in water was heated to 98 °C for 5 min and then immediately placed on ice. After the addition of cold 5x CSB (final concentration 1x), the RNA was incubated on ice for at least 20 min before it was used for the measurement. All solutions were equilibrated to room temperature before use. The experiments were performed with a MicroCal PEAQ-ITC from Malvern Panalytical (Malvern, UK). The cell of the instrument was filled with 200 µL of buffered RNA solution and the syringe was loaded with 40 µL of buffered LFX solution. After initially setting the temperature to 25 °C and equilibration of the system, the measurement was started with a 60 s delay and an initial test injection of 0.2 µL in 0.4 s. The experiment was then continued with 13 injections of 2 µL in 4 s each. The individual injections were separated from each other by a 150 s delay. Stirring was carried out continuously at 750 rounds per minute (rpm) to ensure an even distribution of the injected ligand. The measurement results were determined in a graph of the change in differential power (DP; µW) over time (min). By integrating the heat ΔQ (µJ; area of each peak) and relating it to the molar ratio of ligand and RNA, the molar enthalpy ΔH (kJ/mol) was calculated. The plot of ΔH against the molar ratio subsequently resulted in a binding curve. If binding was detected, the dissociation constant (*K_d_*) was determined from the fitted binding curve data using the one-site binding model provided by MicroCal PEAQ-ITC Analysis Software (version 1.41). All ITC measurements were performed at least two times.

### 2.5. In-Line Probing

For in-line probing, RNA was prepared as described above. The aptamer was extended with a 21 nt long spacer (5’-GGGCAACAACAACAACAACAA-3’) at the 5’ end to improve the resolution of the cleavage pattern closer to the 5′ end of the aptamer. This spacer was designed to show no interaction with the actual aptamer sequence. Dephosphorylation was carried out with an alkaline phosphatase (Alkaline Phosphatase, Roche) at 50 °C for 30 min. After heat inactivation for 3 min at 95 °C, the RNA was used directly for 5’-labelling. Labeling was performed using the T4 polynucleotide kinase (Roche) with [γ-^32^P]-ATP (6000 Ci/mmol, 10 mCi/mL; HARTMANN ANALYTIC) and incubation at 37 °C for 40 min. Afterward, the sample was purified using a Sephadex G25 column. In-line probing reactions were prepared by mixing 1 pmol of non-labeled RNA, 50 kCPM of labeled RNA with 2x of in-line buffer (40 mM MgCl2, 100 mM Tris HCl, and 200 mM KCl; pH 8.3), and 10X of LFX stock solution. After incubation at room temperature for 4 days, the reaction was stopped by adding 2x of RNA loading buffer (deionized formamide, 25 mM of EDTA, and bromophenol blue) and freezing at −20 °C. The T1 RNase marker and alkaline digestion marker were obtained by using 1 pmol of non-labeled RNA together with 50 kCPM of labeled RNA. For the digestion with T1 RNase, 1 U of the enzyme was added and incubated for 5 min at 55 °C. Alkaline treatment was conducted with 10X of Na_2_CO_3_ solution (0.5 M Na_2_CO_3_, 10 mM EDTA; pH 9.0) for 6 min at 98 °C. In addition, a non-reacted RNA sample was prepared. All samples were mixed with 2x of RNA loading buffer and then separated using denaturing polyacrylamide gel electrophoresis (10% Rothiphorese 40 (19:1, Roth [Karlsruhe, Darmstadt]), 8 M urea). Subsequently, the gel was dried on a heated vacuum bed and analyzed via phosphoimaging using a Typhoon biomolecular imager (Amersham).

### 2.6. Preparation of Hybridization Chain Reaction

Sequence designs for the HCR were based on previously published studies [30,33]. All ssDNA oligonucleotides were ordered from Sigma-Aldrich with HPLC purification; the sequences are given in Table 1. Hairpin 1 (H1), biotinylated Hairpin 1 (H1b), Hairpin 2 (H2), and biotinylated Hairpin 2 (H2b) were diluted to 3 µM in SPSC buffer (150 mM Na_2_HPO_4_, 750 mM NaCl). They were heated to 95 °C for 5 min and slowly cooled to room temperature to allow the formation of the secondary hairpin structures. The Capture Initiator (CI) was diluted to 1 µM with SPSC buffer. For HCR, three equal volumes of an H1 and H2 solution were combined with six equal volumes of H1b and H2b, after which nine equal volumes of CI were added. The reaction was then left at room temperature overnight.

### 2.7. Preparation of Lateral Flow Assays

The design of the assay was adapted from a previous study by Ying and colleagues [30]. A Streptavidin Gold Conjugate suspension (40 nm 10 OD, Abcam [Cambridge, UK], ab186864) was diluted 4-fold with SSC buffer + 4% BSA (15 mM Na-citrate, 150 mM NaCl, and 4% *w*/*v* BSA). Glass Fiber Conjugate Pads (EMD Millipore [Billerica, MA, USA], CFCP203000) were cut into 5 by 5 mm squares and infused with 20 µL of diluted gold nanoparticle suspension each. The pads were then dried at 30 °C for 1 h. The prepared HCR batch was diluted 18-fold in SSC buffer with 4% BSA and additional glass fiber pads were infused with 20 µL each and dried, as above. The nitrocellulose membrane (UniSart CN 95 Polyester backing 100 µm, Sartorius Stedim [Göttingen, Germany], 1UN95ER100025NT) was cut into 100 by 25 mm large pieces. Both the test and control bands were applied lengthwise with a modified Hyrel 30M 3D printer that held a 100 µL Hamilton syringe that was actuated using a threaded rod and electric motor. The syringe was equipped with a curved, blunt cannula. The syringe was lowered until the cannula barely touched the nitrocellulose membrane. It was then programmed to travel horizontally at a fixed vertical position while dispensing at a rate of 1 µL/cm. For the test band, a solution of Anti-Fluorescein-AP fragments (150 U, Roche [Basel, Switzerland], REF 11426338910) was diluted 10-fold in ultrapure water and applied to the membrane. For the control band, an Anti-Rabbit IgG (whole molecule)-Biotin antibody solution (produced in goat, SIGMA, B8895) was diluted 10-fold in ultrapure water and applied to the membrane at a distance of 10 mm parallel to the test band. The 100 by 25 mm nitrocellulose sheets were then cut into 3 by 25 mm large strips. Cellulose Fiber Sample Pads (EMD Millipore, CFSP223000) were cut into 5 by 30 mm large rectangles. Strips were assembled as follows: Nitrocellulose strips were placed on commercial adhesive tape and secured in place. On one end, first, a gold nanoparticle containing a conjugate pad and then a pad containing the HCR reaction mix were placed on the strip. On the other end, cellulose pads were placed as an absorbent pad.

For the hybridization prior to analyte addition, 25 µL of 1x CSB containing 150 pmol of aptamer and 0.5 pmol of 6FAM labeled Capture-ON were heated to 65° for 5 min and left to cool to room temperature. Further, 25 µL of 1x CSB containing twice the final concentration of analyte (LFX or CFX) were added, to reach a total of 50 µL with the desired analyte concentration. After a 2 min incubation at room temperature, the solution was slowly applied to the pad containing the HCR reaction mix. After 20 min, all pads were removed to stop further migration of the liquid and the nitrocellulose strips were dried at room temperature. Each experiment was repeated five times. Please see the Supplementary Materials, Figure S4, for a detailed schematic of the LFA preparation.

Dried nitrocellulose strips were imaged using a ChemiDoc MP Imaging System (BioRad, Hercules, CA, USA) set to white light monochromatic image acquisition (Settings: “No Filter”, “White Epi Illumination”). All strips were photographed in the same image to ensure uniform exposure of the camera for all bands. The image was exported in TIFF format and analyzed in FIJI [38]. Each strip was selected as a separate lane using the Analyze > Gels > Select Lanes function. After selection of all strips the Analyze > Gels > Plot Lanes function was used. The peaks of the resulting plot were selected using the “Wand” tool to obtain the area under the peak.

### 2.8. RNA Capture-SELEX and Next-Generation Sequencing

RNA Capture-SELEX was performed for the selection of an LFX-binding RNA aptamer using a pool design that we had successfully applied before for the selection of small-molecule-binding aptamers [19,20]. The pool contained a 13 nt long docking sequence which was located between two randomized regions that were 50 nt and 10 nt long. The randomized regions were flanked by two constant regions to allow PCR amplification [20]. A library with 10^15^ different RNA sequences was created as a starting pool for the first selection round. A radioactive tracer [ɑ-^32^P]-ATP was incorporated into the RNA sequences to monitor the progression of the selection. For the first round, 1 mL of magnetic bead suspension was used to immobilize the whole library of 10^15^ RNA molecules. About 150 µL of magnetic bead suspension was used for all following cycles. Buffer conditions remained constant for all rounds.

**Figure 1:**
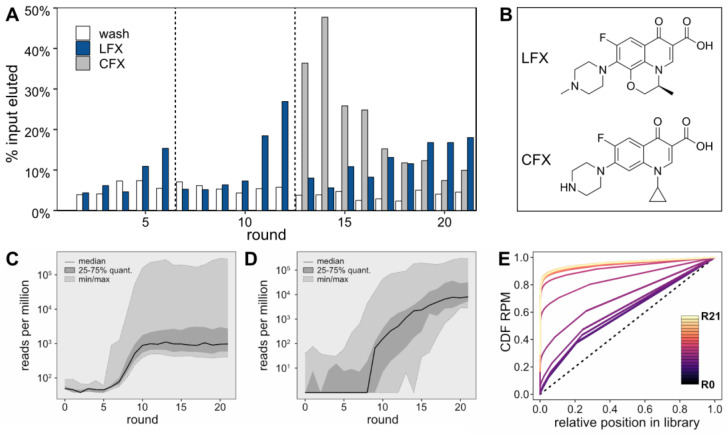
(**A**) RNA Capture-SELEX with LFX as a target molecule. The white bars represent the amount of RNA eluted in the last washing step. The blue bars show the amount of eluted RNA after the addition of 1 mM (rounds 2–6) and 0.1 mM (rounds 7–21) of LFX. To select RNA sequences that specifically bind LFX, a counter-selection with 0.1 mM CFX was introduced from round 13 onwards. The amounts of RNA eluted in this process are shown with dark grey bars. Dashed lines mark increases in stringency due to a reduction in the LFX concentration (starting round 7) or the introduction of a counter target (starting round 13). The first round was not radioactively labeled. (**B**) Skeletal formulae of LFX and CFX. (**C**) Fluctuating frequencies of the top 100 sequences over all rounds. The frequency is given as reads per million on a log10 scale. For each round, the median frequency is depicted by a solid line, the interquartile range (25th to 75th percentiles) is displayed as a darker grey area, and the range from the minimum to maximum frequency is presented as a lighter grey area. (**D**) Progressive enrichment of the final round’s top sequences across all rounds. This figure illustrates the variability and increasing enrichment of the top 25. Each sequence identified as a top sequence in the final round is tracked for its frequency in every round. The median frequency is indicated by a solid line, the interquartile range as a darker grey area, and the range from minimum to maximum frequency as a lighter grey region. (**E**) Evolution of sequence diversity in rounds 0–21 (R0–R21) using a Lorenz curve. The X-axis represents the abundance rank and the Y-axis indicates the Cumulative Distribution Function (CDF) of RPM. Each colored line represents a different round, with the color gradient moving from darker (R0) to lighter (R21) as the rounds progress. The 45-degree dotted line represents a perfectly even distribution of sequence frequencies, which is used as a reference point against which the actual distribution is compared.

For the initial six selection rounds, a 1 mM LFX solution was used for the selective elution step (Figure 1A, blue bars). In round 6, the LFX-eluted fraction showed about three times as much eluted RNA as the last wash step, indicating an enrichment of the pool. Starting in round 7, the LFX concentration was reduced to 0.1 mM to increase stringency. This initially led to a decrease in the amount of LFX-eluted RNA until round 9. In round 10, the fraction of LFX-eluted RNA sequences started to increase again. This enrichment continued in rounds 11 and 12, after which a counter-elution step with a 0.1 mM solution of the closely related compound CFX was introduced to select for LFX-specific binders (Figure 1A, grey bars). This counter-elution step was performed right before the selective elution step with a 0.1 mM LFX solution. Remarkably, the counter-elution fraction largely exceeded the LFX-eluted fraction up to round 16, indicating that the prior enrichment was less specific and contained CFX binding aptamers. However, starting from round 19, the LFX-eluted fraction rose above the CFX-eluted fraction. The skeletal formulae of LFX and CFX are provided in Figure 1B. All sequencing data were deposited to NCBI’s Sequence Read Archive (Accession: SAMN39271360).

For each round, the sequences of the LFX-eluted fraction were recovered and sent for NGS. The results were subjected to thorough bioinformatics analysis, which facilitated the elucidation of intricate patterns and behavior within the sequence pool. As can be seen in the Supplementary Materials, Figure S3, the sequencing quality was high, with the expected error for the majority of sequences being near zero, indicating reliable data for analysis. When evaluating the RPM of the 100 most abundant sequences in each round of selection (Figure 1C), the initial five rounds did not demonstrate any enrichment of consistent sequences. However, a noticeable shift occurred between rounds 6 and 10, where a significant increase in the abundance of the top sequences from each individual round was observed, culminating in a stable high plateau that persisted until the final 21st round. This surge in sequence abundance closely aligns with the heightened stringency in the Capture-SELEX process, suggesting that increased selection pressure led to the dominance of these top-performing sequences. The enrichment pattern of the most abundant 25 sequences from the final round varied throughout the selection process (Figure 1D). The median began to rise after the 7th selection round, which is before a considerable enrichment could be observed in Figure 1A. Finally, an analysis of the cumulative distribution function (CDF) of RPM was performed (Figure 1E). This analysis focused on the abundance of all sequences in the RNA pool, ranked from least to most frequent. Beginning with round 0 it was found that, although the starting pool of the selection process did not exhibit a perfectly uniform distribution (represented by a diagonal line in the CDF plot), it was reasonably close to it. The CDF of the starting pool shows little change until round 6. However, between rounds 6 and 11, considerable shifts in the CDF are observed, indicating a notable enrichment of specific sequences. This level of enrichment continues to remain high from round 12 onwards.

### 2.9. Analyte Induced Release of the Top Eight Aptamers

The eight most abundant sequences in round 21 were determined from NGS data (Table 2). Sequences A1 and A2, as well as B1 and B2, only differed by one nt, respectively. Their abundances are therefore given as the sums of both sequence variants. The most abundant sequence was E (293,779 RPM) making up 29% of the sequences in round 21. Overall, the top 8 sequences made up 55% of round 21.

**Figure 2:**
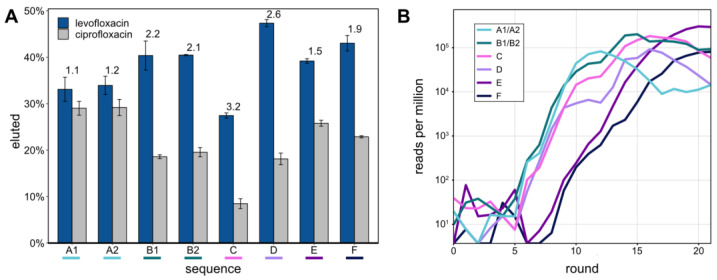
(**A**) Elution assay under Capture-SELEX conditions. Eight different potential LFX binding aptamers, designated A–F, were tested (sequences designated with a number only differ by one point mutation, see Table 2). The elution was tested independently for either LFX or CFX. Blue bars represent the percentage of RNA eluted upon the addition of 1 mM of LFX. Light grey bars represent the amount of RNA eluted with 1 mM of CFX. The colors under the sequence names correspond to the colors used in panel B. The experiment was repeated three times; standard deviation is given. Ratios of the eluted LFX and CFX fractions are indicated above the bars. (**B**) Individual tracing of sequences A–F through all rounds. The Y-axis denotes the RPM, shown on a logarithmic scale.

These top 8 aptamers were tested for their capacity to bind LFX and CFX and subsequent release from the Capture-ON. This binding of the aptamer to the Capture-ON mimicked the hybridization step throughout the performed Capture-SELEX. After immobilization on magnetic beads, the aptamers were separately tested for elution with 1 mM of LFX or CFX, respectively (Figure 2A). All of the eight aptamers demonstrated robust release from the Capture-ON after incubation with LFX, though their capacity to distinguish LFX from CFX varied. The specificities of the tested aptamers were quantified with the ratios of the LFX-eluted fractions and CFX-eluted fractions (indicated above the bars in Figure 2A). The aptamers LxA1 and LxA2, which only differed in one nucleotide, showed highly similar release after incubation with LFX and CFX. The same is true for sequences LxB1 and LxB2. However, aptamers LxB1/2 showed a slightly higher release than aptamers LxA1/2 while also distinguishing LFX and CFX much better with ratios of 2.2 and 2.1. Aptamer LxC had the lowest LFX released fraction at 27.4% but showed the highest specificity of the eight aptamers with a ratio of 3.2. Aptamer LxD showed the highest release with LFX at 47.3% and the second-highest specificity ratio of 2.6. Aptamers LxE and LxF had a similar release as aptamers LxB1/2 though with lower specificity ratios.

Using the obtained NGS data, all eight aptamers were traced throughout the SELEX rounds (Figure 2B). Aptamers A to D started enriching after round 5 in a similar pattern, which correlates with the first enrichment of the radioactively labeled elution fractions (Figure 1A). Aptamers E and F started to enrich after round 8, which links to the second enrichment that was observed after the stringency of the selection was increased by changing the LFX concentration of the elution step from 1 to 0.1 mM.

### 2.10. Isothermal Calorimetry of Truncated Aptamer Variants

**Figure 3:**
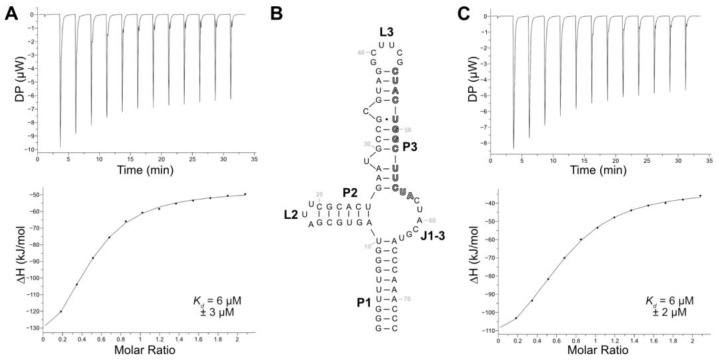
(**A**) Representative ITC thermogram and titration curve of the aptamer LxC titrated with LFX. The dissociation constant derived from two independent measurements and statistical range are given. (**B**) Secondary structure of aptamer LxCsh as predicted using RNAfold [39]. Paired regions (P), loops (L), and the junction (J) are indicated. The capture sequence is marked in bold grey. (**C**) Representative ITC thermogram and titration curve of the aptamer LxCsh titrated with LFX. The dissociation constant derived from two independent measurements and statistical range are given.

Among the eight aptamers that were tested in the release assay, aptamer LxC had the lowest release but demonstrated the highest specificity for LFX. While the sensitivity of a biosensor can be adjusted with signal amplification, the selectivity of the aptamer fundamentally shapes the specificity of the final biosensor. Therefore, aptamer LxC was chosen for further analysis and optimization. The aptamer sequence still contained both constant regions and had a length of 123 nt. Its secondary structure—as calculated using RNAfold [39]—proposed a closing stem (P1), two stem–loop structures (P2-L2, P3-L3), and a junction connecting all stems (J1-3). Additionally, two bulged-out nucleotides (U29, C34) were predicted in stem P3 (see Supplementary Materials, Figure S5). The binding characteristics of aptamer LxC were determined using ITC, which resulted in a *K_d_* of 6 µM (Figure 3A). Aptamer LxC was then truncated, and the resulting new closing stem stabilized with three G:C base pairs (Figure 3B). The ITC measurements of the shortened aptamer LxCsh showed the same *K_d_* of 6 µM (Figure 3C) and were used for all further truncation experiments.

**Figure 4:**
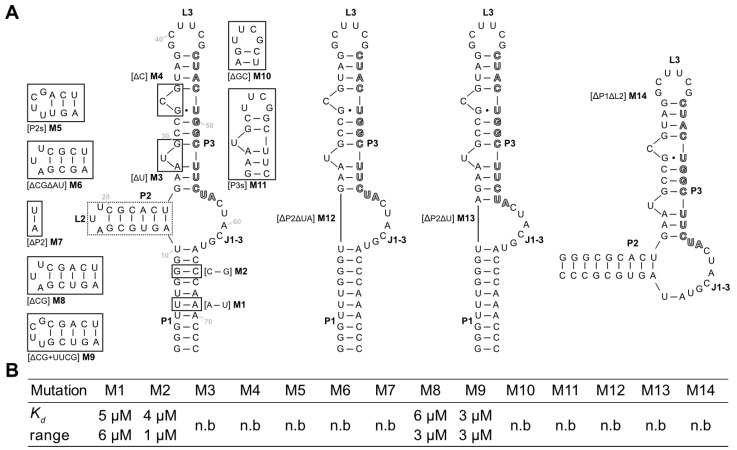
(**A**) Mutation analysis of the aptamer LxCsh. Mutations M1-M14 are displayed (boxed regions). Stems (P), loops (L), and the junctions J1-3 are marked. The capture sequence is highlighted in bold grey. (**B**) The results of the binding studies of the mutants performed via ITC. *K_d_*s of the mutants are shown. Values are measured twice; statistical ranges are given. An “n.b.” indicates that no binding could be observed in the ITC experiment.

Next, mutations and truncation studies were performed to optimize the aptamer size and to determine which parts of the sequence are responsible for ligand binding. A total of 14 variants were designed and tested for LFX binding using ITC (Figure 4A). The results of the binding assays are summarized in Figure 4B, and the full thermograms and titration curves are shown in the Supplementary Materials, Figures S6–S19. The mutations were used to investigate whether shortening of the stems (M5–M9, M11), exchange of the loops (M5, M9, and M10), or deletion and swapping, respectively, of individual bases/base pairs (M1–M4) was possible without losing the ability to bind to LFX.

Of the tested sequences, only four retained their binding capacity. Mutations M1 and M2 which both flipped a base pair of the P1 stem showed no change in binding to LFX. This indicates that the P1 stem does not directly contribute to binding. M5–M9 targeted the P2 stem–loop while retaining A11 and U25 of the junction J1-3. M5, which shortened the stem by 3 base pairs and introduced a stable UUCG tetraloop, lost all binding capacity. The same is true for M6, which shortened the stem by 2 base pairs, and M7, which had the P2 stem removed. In contrast, M8 and M9, which both had just one G:C pair removed, could still bind LFX with no impediment. This hints at the importance of a sufficiently long P2, which is further underlined by the observed loss of binding in M12 and M13, which both had the complete P2 stem removed. M3 and M4 deleted the two bulged-out nucleotides of P3. These deletions led to a total loss in binding. M10 had the apical hexaloop L3 replaced with a stable UUCG tetraloop, which again led to a loss of binding capacity. The same was observed for M11, which contains a shortened P3 stem capped with a UUCG loop. Together with the results of the deletion of the single bulged-out nucleotides (M3, M4), this suggests that P3 has a crucial role in binding LFX. Finally, M13 had the complete P1 stem removed but retained U10 and A64 of the junction. Here, too, no binding could be observed. This demonstrates that P1 is important for the aptamer, even though it can tolerate mutations (M1, M2).

### 2.11. In-Line Probing

**Figure 5:**
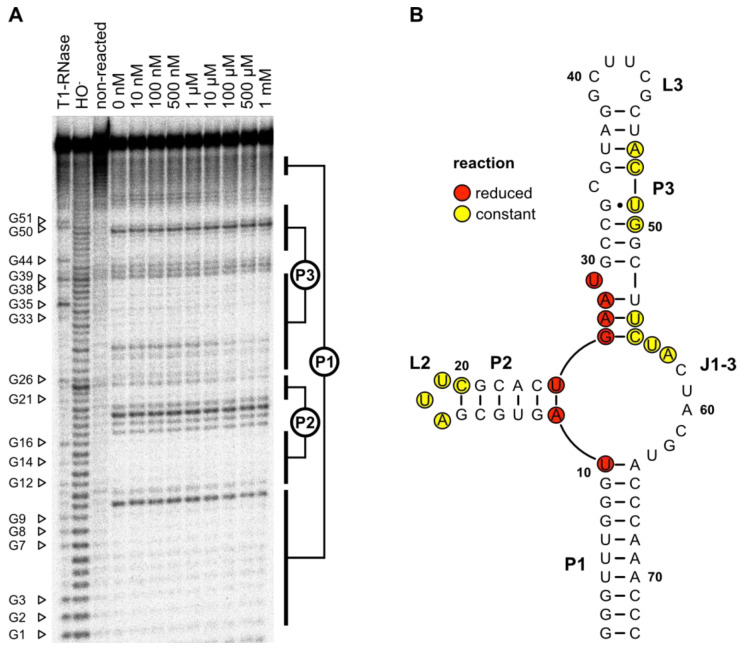
(**A**) In-line probing experiment for LxCsh. The cleavage patterns from 0 to 1 mM LFX are shown. Nuclease T1 digestion (T1-RNase) and hydroxyl reaction (HO−) were loaded onto the gel as references for the determination of the nucleotide positions. G-nucleotides are marked with their position. In addition, a non-reacted RNA sample of the aptamer was included. Likely paired regions are indicated with the corresponding labels (P1, P2, and P3). (**B**) Predicted secondary structure of LxCsh. Nucleotides that showed reduced reactivity as the LFX concentration increased are marked in red. Those that showed constant cleaving independent of the LFX concentration are marked in yellow.

In-line probing was performed to evaluate whether the predicted secondary structure and the results from the mutation experiments could be proven by structure-dependent RNA cleavage (Figure 5A). The cleavage pattern confirmed the predicted secondary structure. The 10-base pair long closing stem P1 and the 6-base pair large stem P2 with its triple loop L2 could be clearly identified. Furthermore, the P3–L3 stem–loop and the bulged U29 could be detected. No signal was detectable at the predicted bulged-out nucleotide C34. The junction J3-1 between the stems P3–P1 also showed detectable cleavage.

The addition of an increasing concentration of LFX led to a more rigid structure of the J1-3 junction at the base of stem P2. U10 and A11 as well as the region from U25 to U29 showed less cleavage in the presence of LFX, indicating that these regions are affected by ligand binding. All LFX-mediated changes in the cleavage pattern are highlighted in red in the 2D structure displayed in Figure 5B.

### 2.12. Modular Lateral Flow Assay

**Figure 6:**
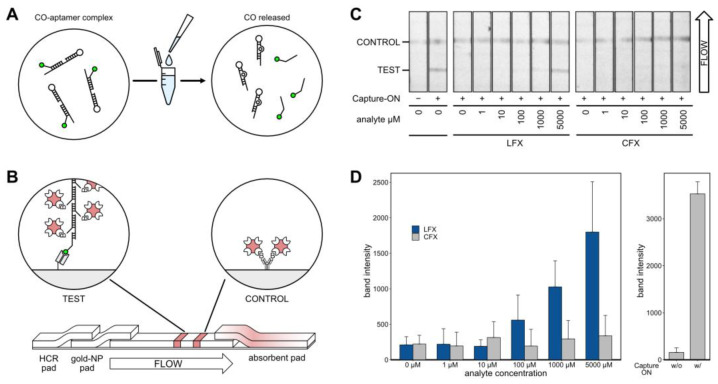
(**A**) The analyte is added to the hybridized Capture-ON/aptamer complex. Upon analyte binding, the Capture-ON is displaced. (**B**) A solution containing the freed Capture-ON and analyte is put on the lateral flow strip. Capture-ON binds to a complementary adapter sequence that connects to the hybridized chain of biotinylated DNA oligonucleotides. When passing through the respective glass fiber pad, streptavidin-conjugated gold nanoparticles are picked up by the biotin residues. Capture-ON with the associated gold nanoparticles is caught by an anti-fluorescein Fab fragment at the test line. A flow control line catches streptavidin-conjugated gold nanoparticles. (**C**) Representative results of the LFA. Images were taken with a monochromatic camera to allow for uniform quantification of the band intensities. Control and test lines are labeled and the direction of flow is indicated. The tested analyte and respective concentrations are given below. The two leftmost strips show controls without any analyte and aptamer, with and without free Capture-ON present. (**D**) Quantified band intensities from 5 independent experiments. Intensities were measured using FIJI [38]; standard deviation is given. The left plot shows experiments comparing responses to LFX and CFX. The right plot shows the respective controls without and with added Capture-ON.

After thorough characterization of the aptamer, it was incorporated into an LFA for visual detection of LFX (Figure 6). The LFA was based on the displacement of the Capture-ON upon binding of LFX. Prior to analysis, the aptamer LxCsh was hybridized with the Capture-ON by heating and slowly cooling down to room temperature. After the addition of the analyte, the labeled Capture-ON was released upon the binding event (Figure 6A). Next, the solution containing the bound aptamer and the released Capture-ON was placed on a lateral flow strip (Figure 6B). The solution was loaded on a dried glass fiber pad containing a hybridized chain of biotinylated DNA oligonucleotides. This hybridized chain contained an adapter sequence that was complementary to the Capture-ON sequence. The solution was then passed into an adjacent glass fiber pad holding streptavidin-conjugated gold nanoparticles that attached to the biotinylated chain of oligonucleotides. Thereby, a single Capture-ON was connected to multiple gold nanoparticles, which served as signal amplification for visual detection. The solution then traveled through a polyester-backed nitrocellulose membrane and passed two prepared lines (Figure 6B). First, the test line consisted of immobilized anti-6-FAM Fab fragments. These Fab fragments caught Capture-ONs with the attached DNA chains and gold nanoparticles, allowing for a red visual readout in the detection of LFX. The second control line consisted of unspecific, biotinylated IgG antibodies. These biotins caught excess gold nanoparticles and functioned as a flow control. Of note, the IgG antibodies did not serve a purpose aside from presenting biotin. In testing, it was found that the nitrocellulose membrane bound to the IgGs very well; however, no other proteins were tested. Both lines were prepared using a modified Hyrel 30M 3D printer that held a 100 µL Hamilton syringe, allowing for the precise application of anti-6-FAM Fab and biotinylated IgG solutions. At the end of the nitrocellulose strip, a cellulose pad was placed to absorb and pull the solution through the strip.

Exemplary results of the LFA experiments are shown in Figure 6C. All experiments were repeated five times and strips were imaged using a ChemiDoc MP Imaging System (BioRad) set to white light monochromatic image acquisition. All strips showed a visible flow control. In the presence of the aptamer but in the absence of the analyte and Capture-ON, no test band appeared. Positive control with Capture-ON but without aptamer showed a clearly visible band. Experiments with all components and varying concentrations of analyte showed an increasing band intensity corresponding to increased LFX concentrations. While CFX also produced visible bands, the signal was much lower.

Band intensities of five independent LFA experiments were analyzed using FIJI [38]. The results are depicted as bar charts in Figure 6D; standard deviations are given. When testing LFX concentrations, the LFAs showed bands with increasing intensity at 100 µM and above, while CFX leads to very little discernable signal, even at 5000 µM. The band intensities of control experiments are given on the right of Figure 6D.

## 3. Discussion

This study demonstrates how RNA Capture-SELEX can be employed for the development of an LFX-binding RNA aptamer and its use in a biosensor. It shows how the displacement of a Capture-ON can not only be used for the selection of specific aptamers but also as a general mechanism of biosensing in aptamer-based LFAs. The presented workflow serves as a potentially transferrable method for the selection and use of further RNA aptamers as bioreceptors. An LFX-binding DNA aptamer was previously reported and used to develop a very capable biosensor [40]. However, the readout of this DNA-based sensor was fluorescence-based and required laboratory equipment. This study focused on RNA because RNA can potentially form more complex and intricate three-dimensional structures. Consequently, for small molecules, a selection with RNA can result in better aptamers. Moreover, an RNA biosensor may also be used in cells.

The RNA Capture-SELEX was performed for 21 consecutive selection rounds to achieve a satisfactory degree of pool enrichment and sufficiently low elution with the counter target CFX. This is a surprisingly large number of selection cycles when compared with our previous RNA Capture-SELEX studies. The paromomycin and tobramycin aptamers were selected within 10 and 12 cycles each [19,20]. The selection of the CFX aptamer, which was also turned into a biosensor, needed only 10 cycles [13,41]. Strikingly, the present SELEX for LFX showed a strong elution with CFX for five rounds after the counter-elution step was introduced. This indicates that a large proportion of the sequence population bound well, or even better to CFX than LFX. This is somewhat surprising as the chemical structures of both compounds are rather similar, with about the same propensity for H-bridges and pi-stacking (Figure 1B). Neither is obviously more suited to interaction with RNAs. However, the selected aptamer LxC displayed a good preference for LFX in the elution assay, where more than three-fold of the aptamer molecules were released with LFX compared with CFX (Figure 2A). Also, the final aptamer LxCsh can clearly distinguish CFX from LFX in the lateral flow experiments (Figure 6C,D). As an additional analysis, the NGS data of the LFX selection were searched for motifs and similarities to the CFX aptamer [41]. While some motifs were detected in low frequencies, none of the corresponding sequences were enriched or appeared in meaningful numbers.

A combination of in-line probing and ITC studies of aptamer sequence variants revealed that the structure likely consists of three distinct stems, two of which end in an apical loop. All three stems join into one central junction. The closing P1 stem provides stability for the aptamer, which is necessary for ligand binding. However, its exact sequence is not as important as variants M1 and M2 show. Similarly, P2 can tolerate shortening and loop exchanges—as M8 and M9 demonstrate—but cannot be removed entirely without destroying the aptamer’s capacity to bind LFX. As we observed in aptamers that resulted from previous RNA Capture-SELEX selections, the capture sequence makes up an integral part of the final aptamer sequence and structure [19,20,41]. The P3 stem, which contains most of the capture sequence, appears to be mostly intolerant to changes.

The developed LFA showed good specificity when comparing the signal response from LFX and CFX. The assay had a lower limit of visual detection of 100 µM, which corresponds to 23.1 ng/µL. Wastewater concentrations have been reported to reach as high as 20 µg/L [28], which would be below the sensing capabilities of the presented LFA. This places the presented LFX biosensor at the lower end of reported sensitivities for aptamer-based, antibiotic-detecting LFAs. A comparable biosensor for kanamycin achieved a limit of detection (LOD) of 50 nM [42], and another for oxytetracycline achieved 5 pg/µL [43]. Still, there are examples with comparatively low sensitivity that are still very much functional and practical, such as an ampicillin-detecting LFA with a reported LOD of 185 ng/µL [44]. As presented, the LFA’s LOD is likely limited by the comparatively high *K_d_* of the selected aptamer, which hinders its use as a field-testing device. While modularity and sensitivity of the LFA need to be explored further with higher affinity aptamers, this study has demonstrated how a combined approach of Capture-SELEX and displaced strand detection can be used to develop new antibiotic biosensors.

## Figures and Tables

**Table 1:** DNA oligonucleotides used for HCR.

Name	Sequence (5’ to 3’)
H1	TTAACCCACGCCGAATCCTAGACTCAAAGTAGTCTAGGATTCGGCGTG
H1b	btn-TTTTTTTTAACCCACGCCGAATCCTAGACTCAAAGTAGTCTAGGATTCGGCGTG
H2	AGTCTAGGATTCGGCGTGGGTTAACACGCCGAATCCTAGACTACTTTG
H2b	AGTCTAGGATTCGGCGTGGGTTAACACGCCGAATCCTAGACTACTTTGTTTTTT-btn
CI	AGTCTAGGATTCGGCGTGGGTTAACTACTGGCTTCTA

**Table 2:** Sequences and abundances of the top 8 aptamers found in SELEX-round 21.

ID	Sequence *	RPM **
A1	5′cr-UUUUUC**A**GGCGGCUUAAAAACCAGGAAAGGUGGAGACUUCUACUGGCUUCUAUCCAGCCGGU 3′cr	14,297
A2	5′cr-UUUUUC**U**GGCGGCUUAAAAACCAGGAAAGGUGGAGACUUCUACUGGCUUCUAUCCAGCCGGU 3′cr	
B1	5′cr GUGUAACGGAAGCACAAAGGUGCCUCCUACAGCAGGUCUUCUACUGGCUUCUACUGAUAC**A**CU 3′cr	92,688
B2	5′cr GUGUAACGGAAGCACAAAGGUGCCUCCUACAGCAGGUCUUCUACUGGCUUCUACUGAUAC**G**CU 3′cr	
C	5′cr UUGGGUAGUGCGAUUCGCACUGAAUGCCGCGUAGGCUUCGCUACUGGCUUCUACUACGUACCC 3′cr	58,892
D	5′cr-CUAGUGCCUCAACUAGCCGAACCGUGGUCGUCUUCAUGCCUACUGGCUUCUACGAGAUAAAU 3′cr	14,535
E	5′cr CGUAGUGGACCUGUUUAGGUUUGCAUACGUUAGAUGUCUUCUACUGGCUUCUAAAUCGAACCG 3′cr	293,779
F	5’cr GCAGGUCUGGGAACUCGUUCCCGGCUCCAGCUGGAUUCUUCUACUGGCUUCUAGCCCGACCGG 3’cr	80,736

* Bold nts denote single nt differences in sequences A1/2 and B1/2. 5′ and 3′ constant region (cr) are abbreviated for better readability: 5′cr: GGGCAACUCCAAGCUAGAUCUACCGGU; 3′cr: AAAAUGGCUAGCAAAGGAGAAGAACUUUUCACU. Docking sequence is underlined. ** Frequencies are given in reads per million (RPM). For sequences A1 and A2 and B1 and B2, summarized RPM are given.

## Data Availability

The data underlying this article are available in the article and in its online Supplementary Materials.

## Supplementary Materials

The following supporting information can be downloaded at: https://www.mdpi.com/article/10.3390/bios14010056/s1, Supplementary Figure S1: The RNA-Capture-SELEX, Supplementary Figure S2: Bioinformatic workflow of the NGS analysis, Supplementary Figure S3: Boxplot of Expected Error Distribution by File, Supplementary Figure S4: Printing and assembly of the LFA strip, Supplementary Figure S5: Secondary structure of aptamer LxC, Supplementary Figure S6: ITC thermogram and titration curve of the aptamer variant M1, Supplementary Figure S7: ITC thermogram and titration curve of the aptamer variant M2, Supplementary Figure S8: ITC thermogram and titration curve of the aptamer variant M3, Supplementary Figure S9: ITC thermogram and titration curve of the aptamer variant M4, Supplementary Figure S10: ITC thermogram and titration curve of the aptamer variant M5, Supplementary Figure S11: ITC thermogram and titration curve of the aptamer variant M6, Supplementary Figure S12: ITC thermogram and titration curve of the aptamer variant M7, Supplementary Figure S13: ITC thermogram and titration curve of the aptamer variant M8, Supplementary Figure S14: ITC thermogram and titration curve of the aptamer variant M9, Supplementary Figure S15: ITC thermogram and titration curve of the aptamer variant M10, Supplementary Figure S16: ITC thermogram and titration curve of the aptamer variant M11, Supplementary Figure S17: ITC thermogram and titration curve of the aptamer variant M12, Supplementary Figure S18: ITC thermogram and titration curve of the aptamer variant M13, Supplementary Figure S19: ITC thermogram and titration curve of the aptamer variant M14, Supplementary Table S1: Results of 21 rounds and naive pool of merging paired-end reads using VSEARCH’s mergePair function, Supplementary Table S2: Post-Merging Sequence Filtering Results Over 21 Rounds and Naive Pool, and Supplementary Table S3: Number of Unique Sequence Counts Through Preprocessing Stages Over 21 Rounds Plus Naive Pool.
