# Supplementary material for: Sensing Levofloxacin with an RNA Aptamer as a Bioreceptor
Source: Biosensors (Basel). 2024 Jan 22;14(1):56. doi: 10.3390/bios14010056 (PMC10813692; doi:10.3390/bios14010056)
Supplement: Supplementary file 1 [file biosensors-14-00056-s001.zip › biosensors-2775408-supplementary.pdf]

Sensing levofloxacin with an RNA aptamer as bioreceptor

Supplementary Information

Janice Kramat [1,†], Leon Kraus [1,†], Vincent J. Gunawan [1], Elias Smyej [1], Philipp Froehlich [2], Tim E. Weber [3], Dieter Spiehl [3,4], Heinz Koeppl [2,4], Andreas Blaeser [3,4] and Beatrix Suess [1,4,*]

[1] Synthetic RNA Biology, Department of Biology, Technical University of Darmstadt, 64287 Darmstadt, Germany
[2] Self-Organizing Systems, Department of Electrical Engineering and Information Technology, Technical University of Darmstadt, 64283 Darmstadt, Germany
[3] Institute for BioMedical Printing Technologies, Technical University of Darmstadt, 64289 Darmstadt, Germany
[4] Centre for Synthetic Biology, Technical University of Darmstadt, 64289 Darmstadt, Germany
* Correspondence: bsuess@bio.tu-darmstadt.de
† These authors contributed equally to this work.

Supplementary Figure S1: The RNA-Capture-SELEX. P.2
Supplementary Figure S2: Bioinformatic workflow of the NGS analysis. P.3
Supplementary Figure S3: Boxplot of Expected Error Distribution by File P.4
Supplementary Figure S4: Printing and assembly of the LFA strip. P.5
Supplementary Figure S5: Secondary structure of aptamer LxC. P.6
Supplementary Figure S6: ITC thermogram and titration curve of the aptamer variant M1. P.7
Supplementary Figure S7: ITC thermogram and titration curve of the aptamer variant M2. P.8
Supplementary Figure S8: ITC thermogram and titration curve of the aptamer variant M3. P.9
Supplementary Figure S9: ITC thermogram and titration curve of the aptamer variant M4. P.10
Supplementary Figure S10: ITC thermogram and titration curve of the aptamer variant M5. P.11
Supplementary Figure S11: ITC thermogram and titration curve of the aptamer variant M6. P.12
Supplementary Figure S12: ITC thermogram and titration curve of the aptamer variant M7. P.13
Supplementary Figure S13: ITC thermogram and titration curve of the aptamer variant M8. P.14
Supplementary Figure S14: ITC thermogram and titration curve of the aptamer variant M9. P.15
Supplementary Figure S15: ITC thermogram and titration curve of the aptamer variant M10. P.16
Supplementary Figure S16: ITC thermogram and titration curve of the aptamer variant M11. P.17
Supplementary Figure S17: ITC thermogram and titration curve of the aptamer variant M12. P.18
Supplementary Figure S18: ITC thermogram and titration curve of the aptamer variant M13. P.19
Supplementary Figure S19: ITC thermogram and titration curve of the aptamer variant M14. P.20
Supplementary Table S1: Results of 21 rounds and naive pool of merging paired-end reads using VSEARCH's mergepairs function. P.21
Supplementary Table S2: Post-Merging Sequence Filtering Results Over 21 Rounds and naive pool. P.22
Supplementary Table S3: Number of Unique Sequence Counts Through Preprocessing Stages Over 21 Rounds Plus Naive Pool. P.23

Supplementary Figure S1

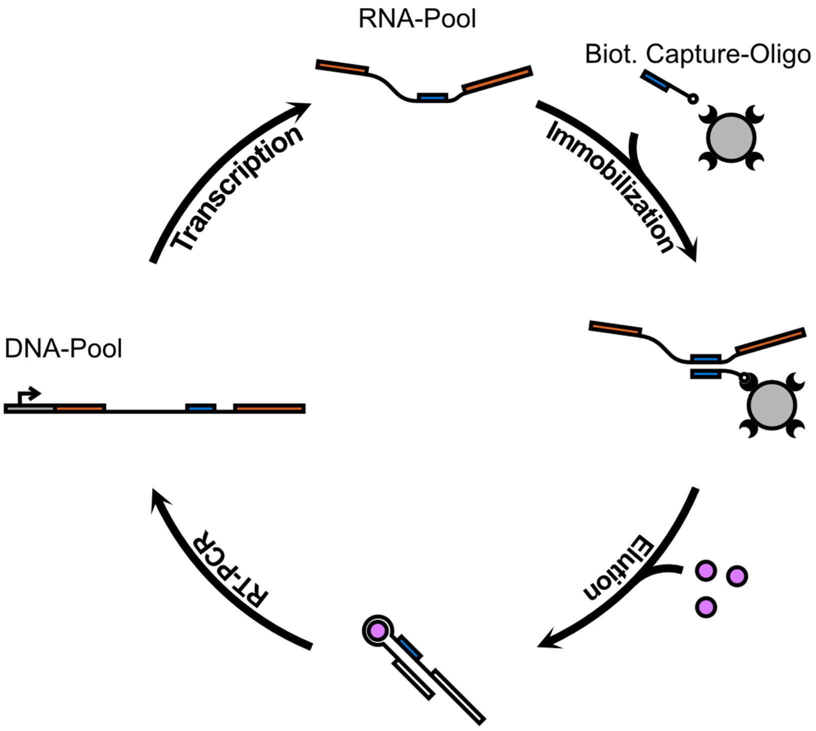

Figure S1. The RNA-Capture-SELEX. Based on Figure 1 in [34]. A randomized DNA-library is transcribed to RNA *in vitro*. The resulting RNA library is hybridized with a biotinylated DNA Capture-ON. The hybridized sequences are immobilized on streptavidin conjugated magnetic beads with the biotin groups of the Capture-ON. A solution containing the target molecule is added. Sequences that undergo a structural change upon ligand binding are eluted. The eluted fraction is recovered, reverse-transcribed and amplified using PCR. The now enriched DNA pool is transcribed again and used for the next cycle.

Supplementary Figure S2

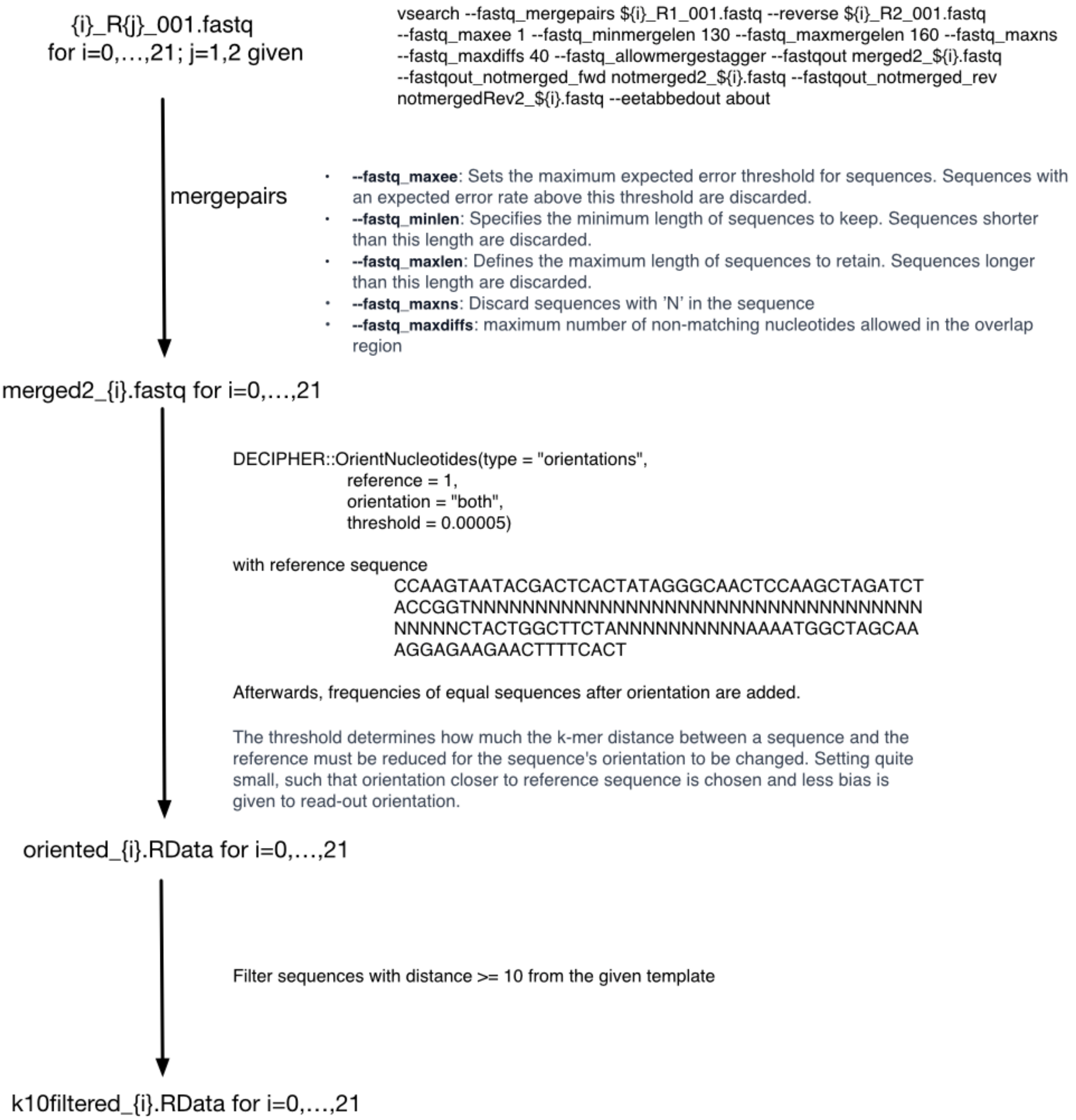

Figure S2. Bioinformatic workflow of the NGS analysis.

Supplementary Figure S3

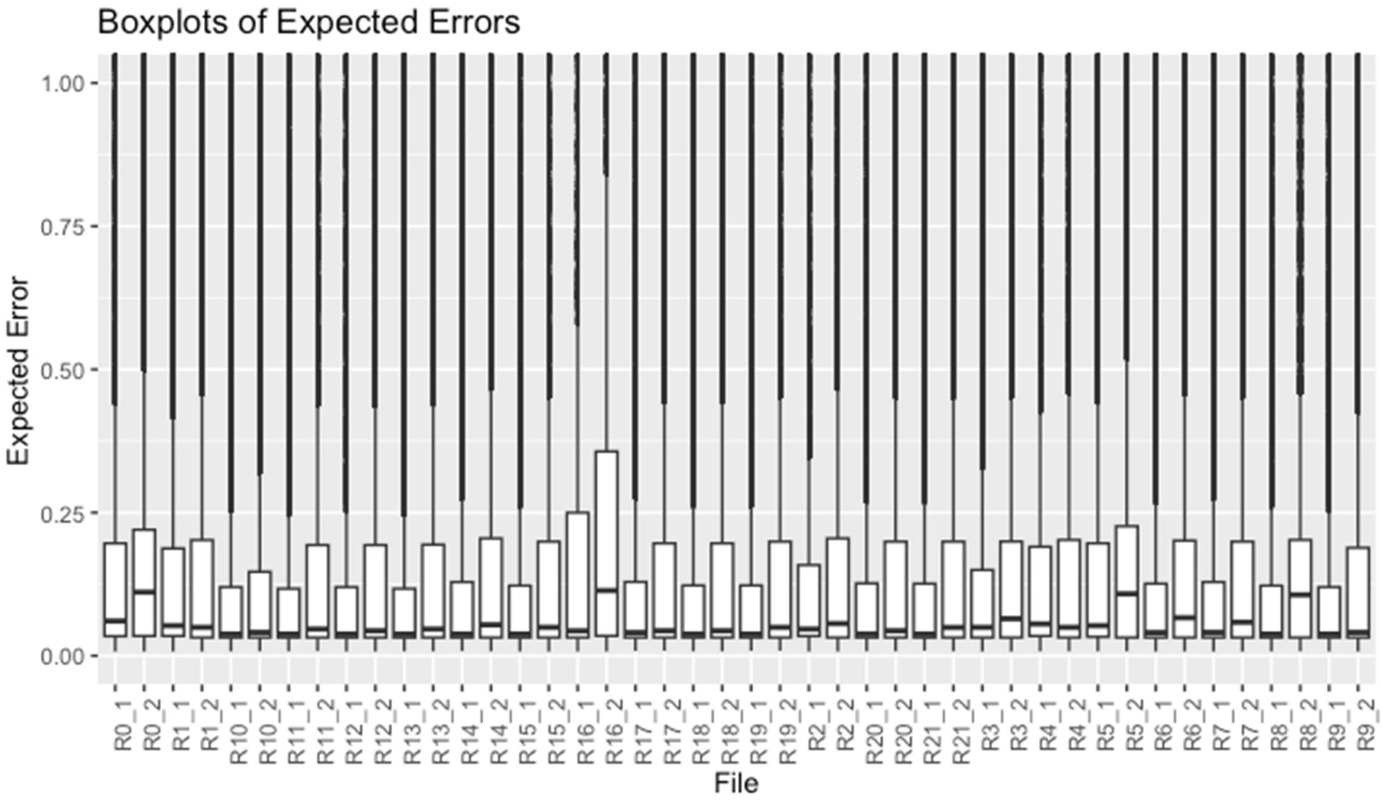

Figure S3. Boxplot of Expected Error Distribution by File. Displays the distribution of expected errors for each sequence derived from sequence quality scores across 22 pools and two orientations. Each boxplot shows the median (central line), interquartile range (box), and 1.5 IQR 'whiskers', with outliers as individual points. This plot provides a succinct overview of sequencing quality across different samples.

Supplementary Figure S4

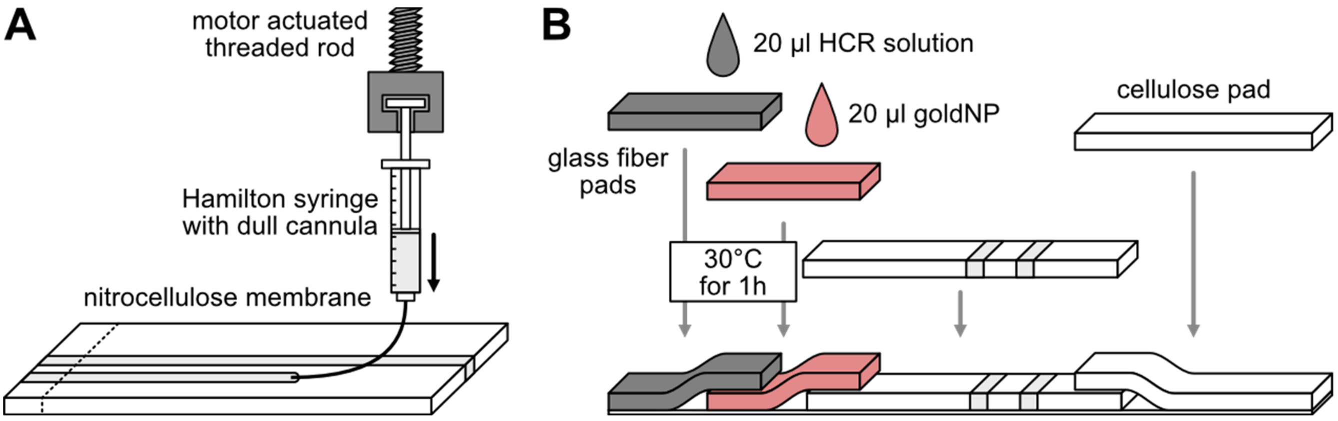

Figure S4. Printing and assembly of the LFA strip. (**A**) Test and control bands were applied to the nitrocellulose membrane using a modified Hyrel 30M 3D printer that held a 100 µl Hamilton syringe that was actuated using a threaded rod and electric motor. Strips were then cut as shown by the dashed line. (**B**) Glass fiber pads were infused with either 20 µl of HCR solution or gold nanoparticle suspension (gold NP) and dried for 1h at 30°C. Glass fiber pads, cut nitrocellulose strip and cellulose pad were then assembled on adhesive tape.

Supplementary Figure S5

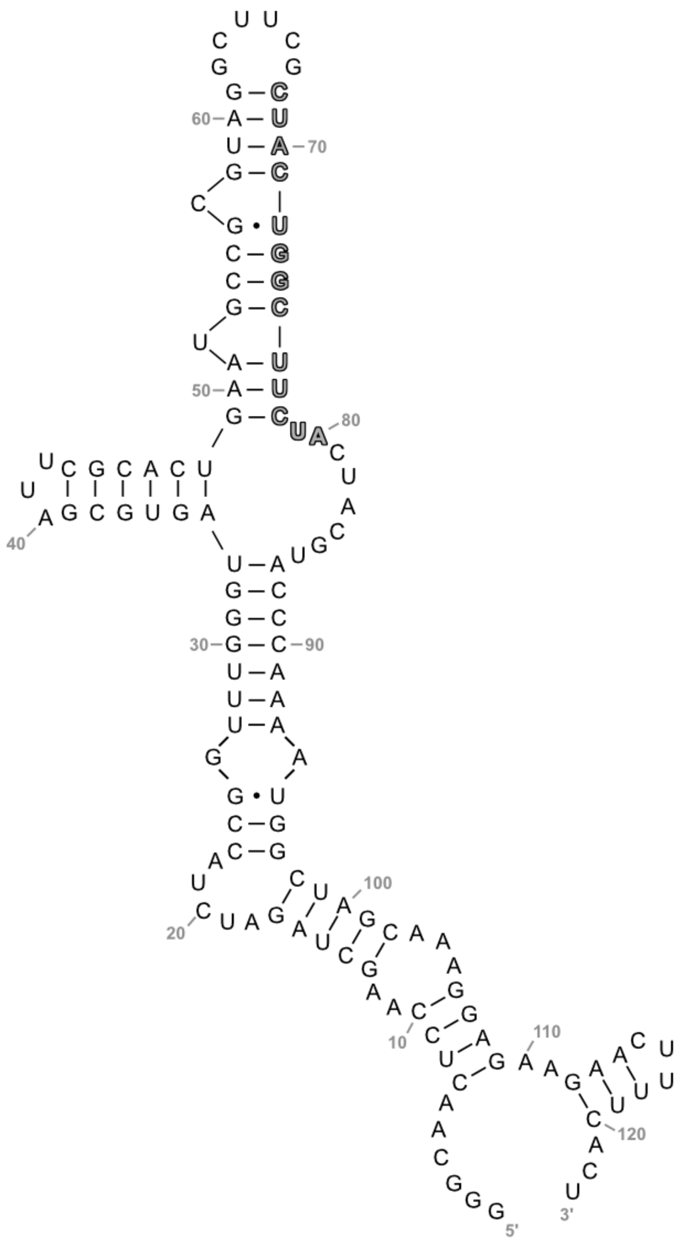

Figure S5. Secondary structure of aptamer LxC. As predicted using RNAfold [39] Capture sequence is marked in bold grey.

Supplementary Figure S6

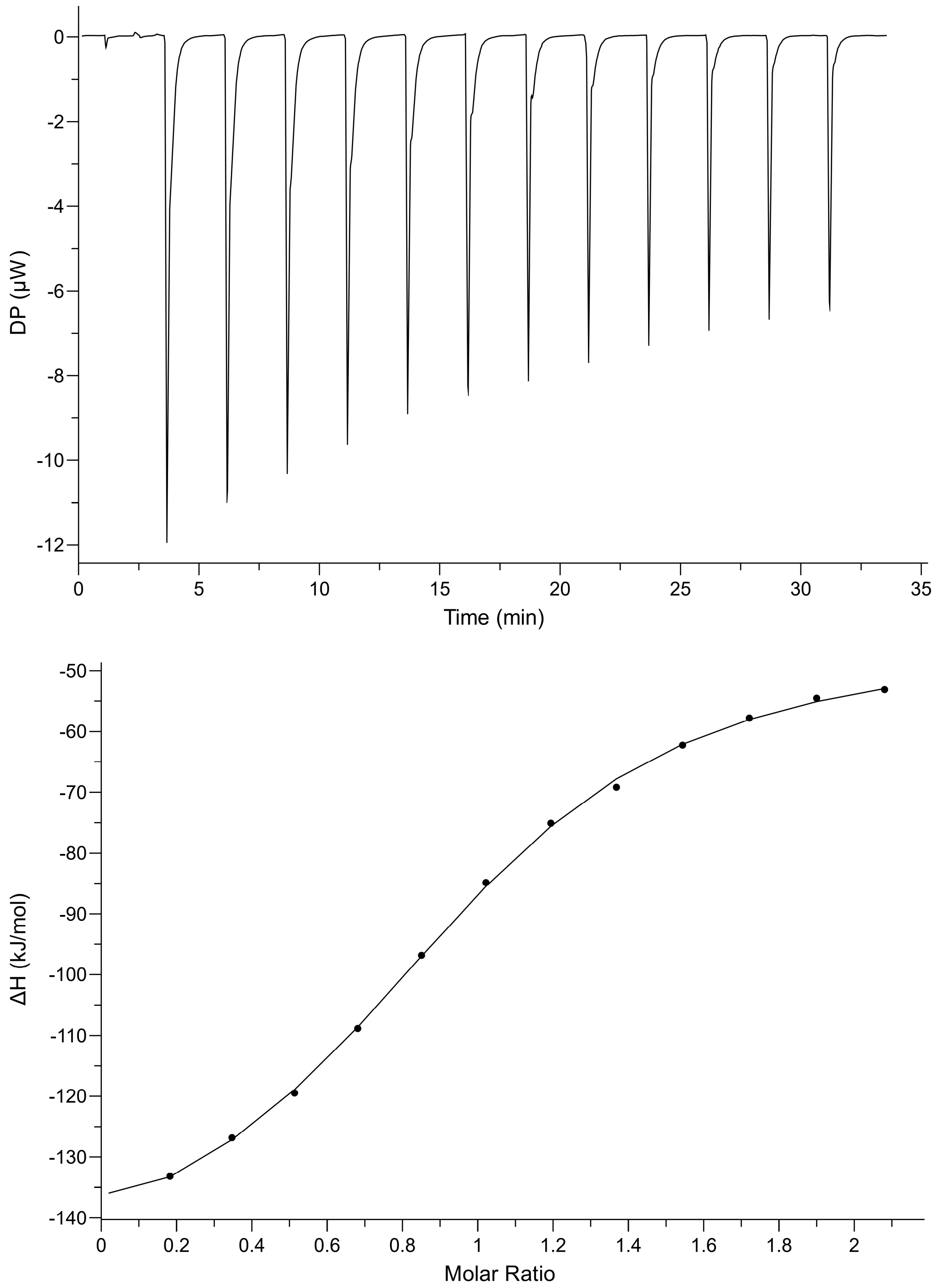

Figure S6. ITC thermogram and titration curve of the aptamer variant M1. Titrated with LFX. Each ITC experiment was repeated at least twice, representative thermogram and titration curve are shown.

Supplementary Figure S7

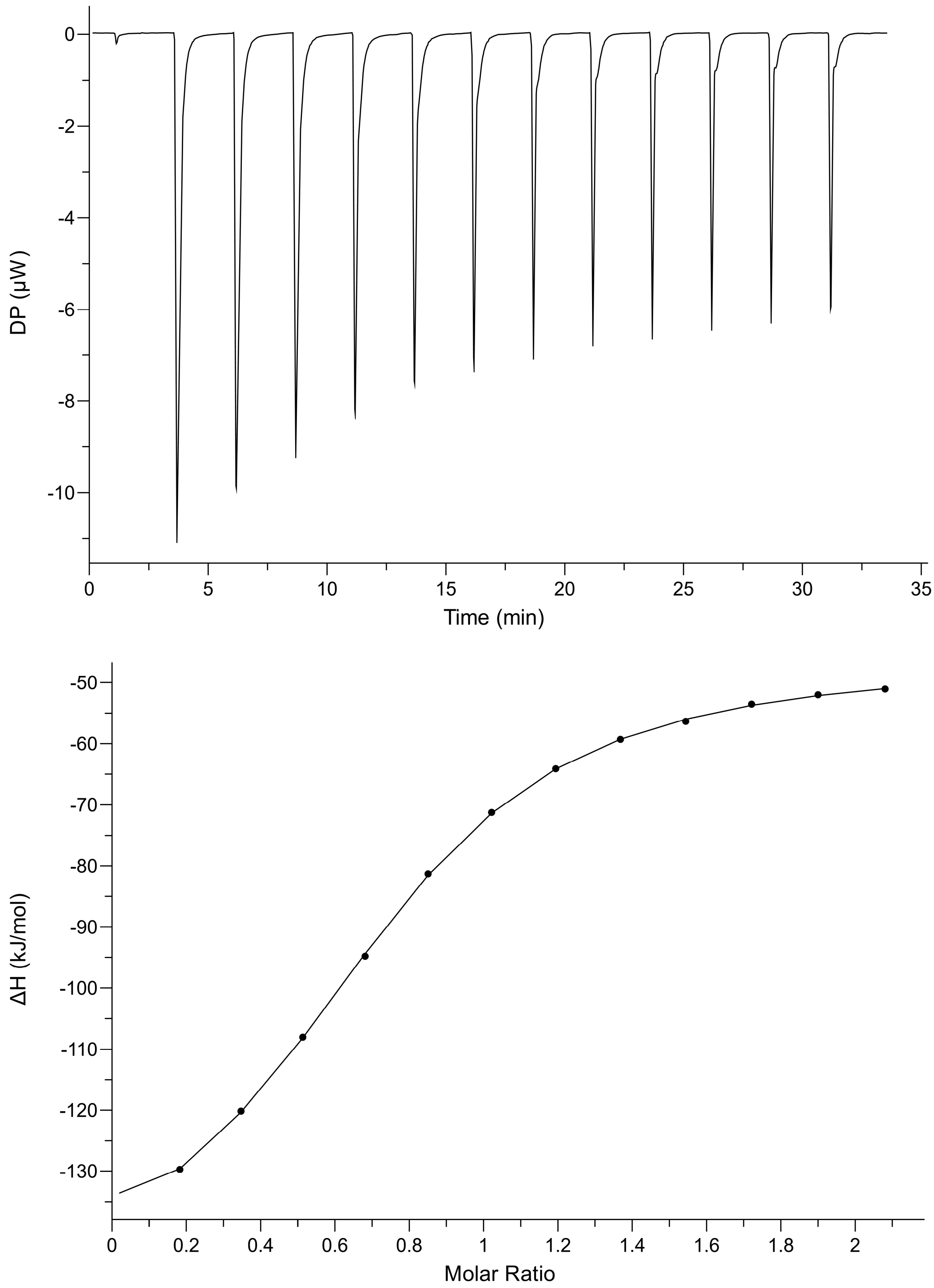

Figure S7. ITC thermogram and titration curve of the aptamer variant M2. Titrated with LFX. Each ITC experiment was repeated at least twice, representative thermogram and titration curve are shown.

Supplementary Figure S8

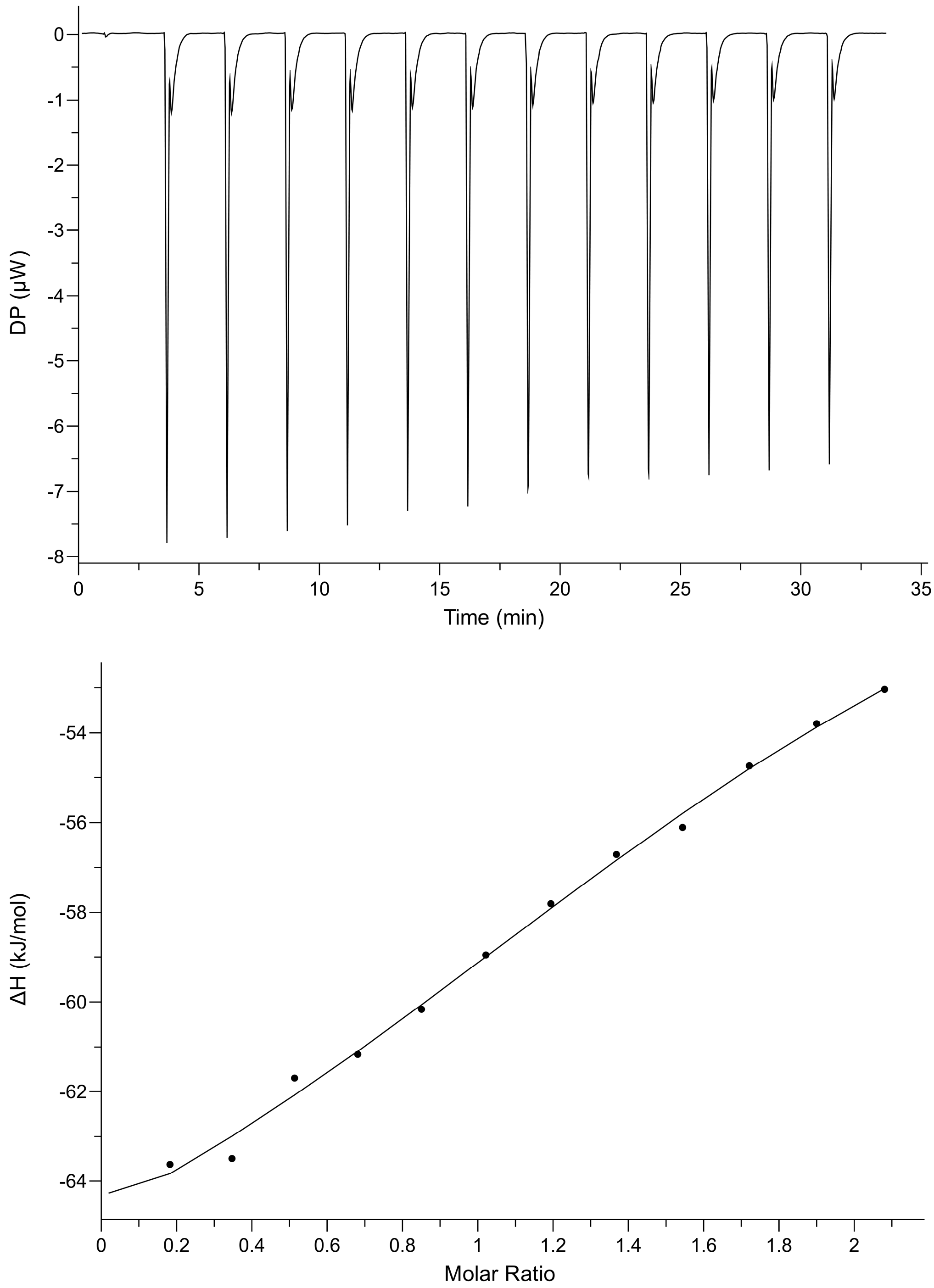

Figure S8. ITC thermogram and titration curve of the aptamer variant M3. Titrated with LFX. Each ITC experiment was repeated at least twice, representative thermogram and titration curve are shown.

Supplementary Figure S9

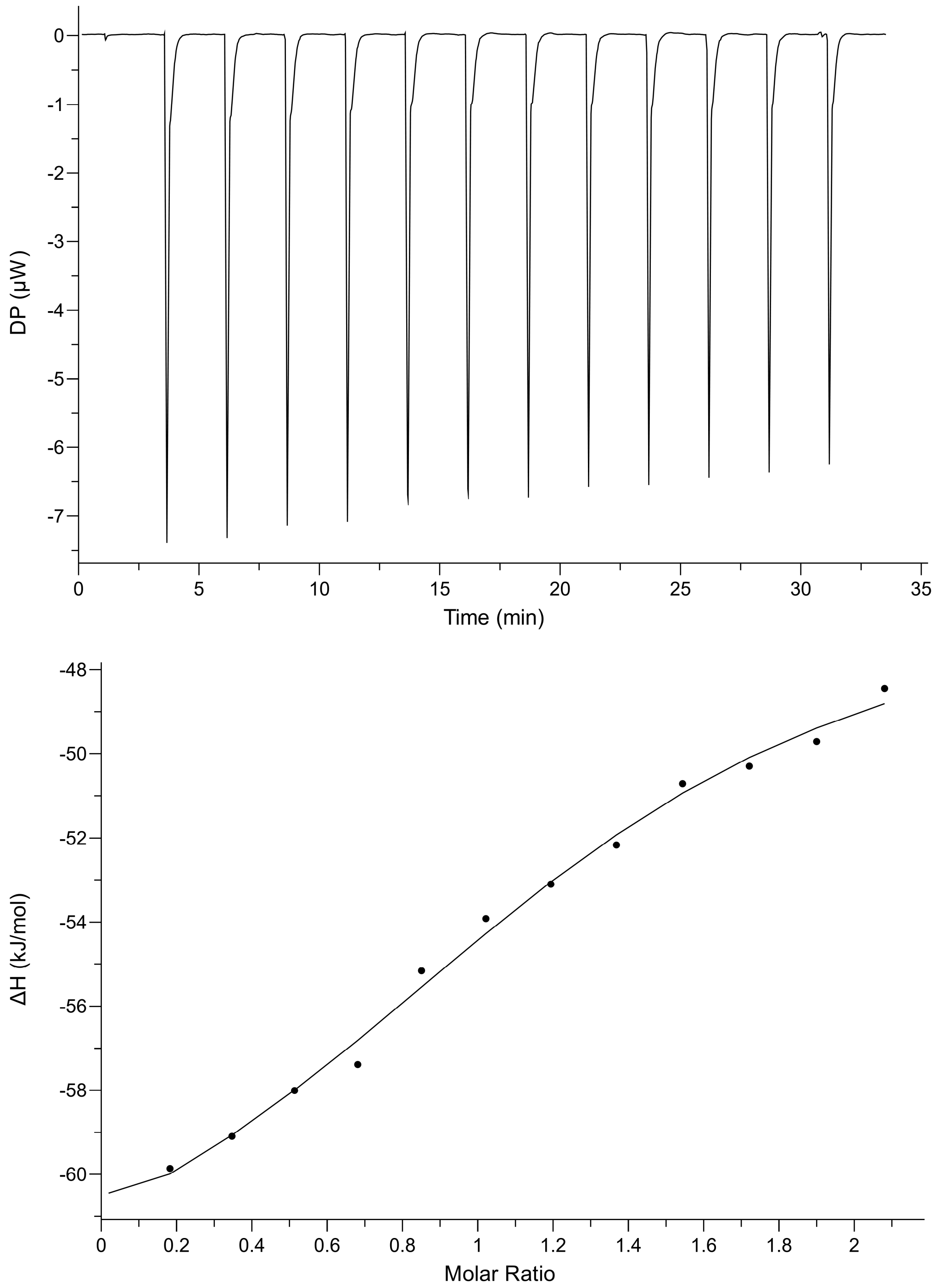

Figure S9. ITC thermogram and titration curve of the aptamer variant M4. Titrated with LFX. Each ITC experiment was repeated at least twice, representative thermogram and titration curve are shown.

Supplementary Figure S10

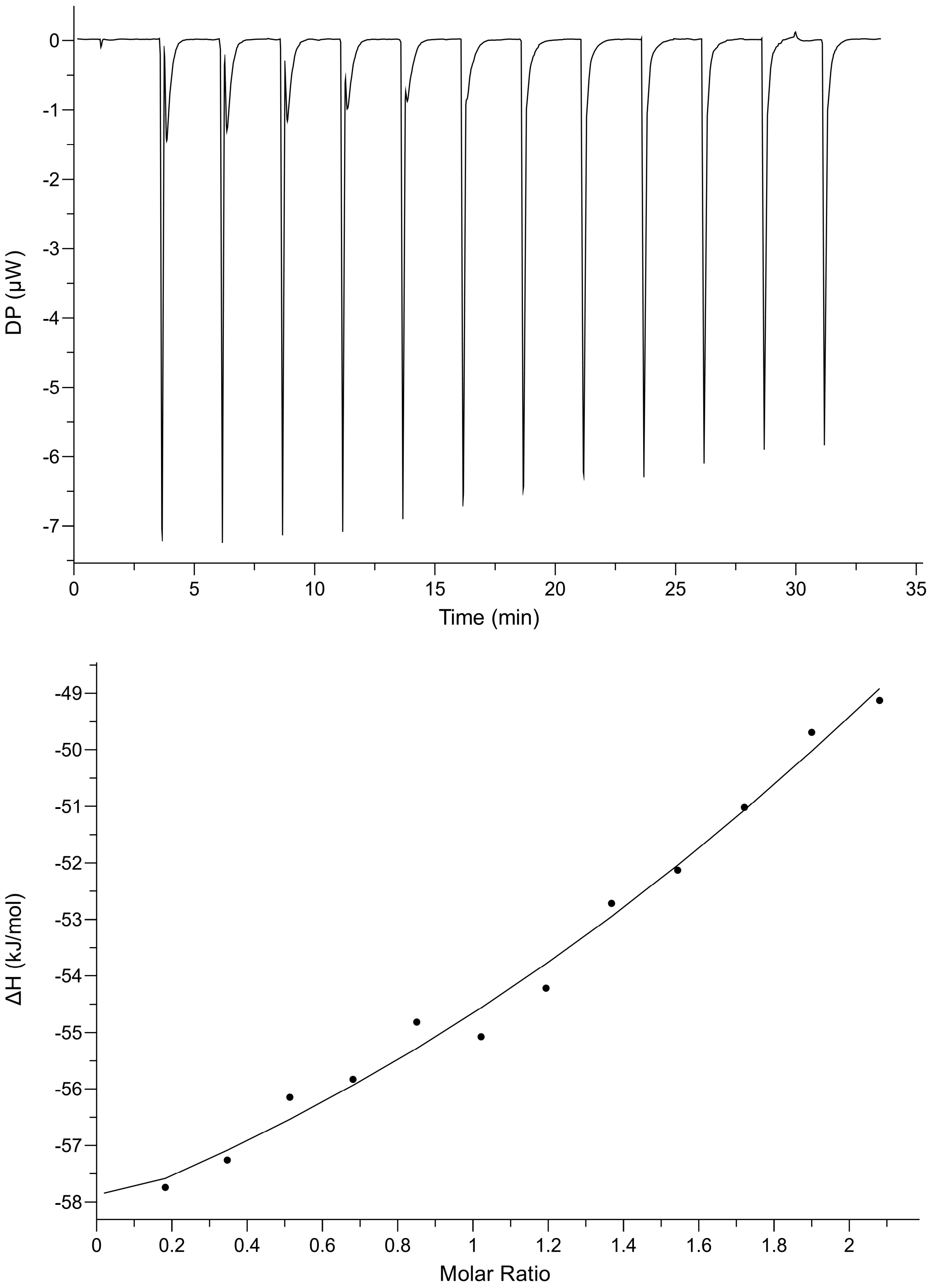

Figure S10. ITC thermogram and titration curve of the aptamer variant M5. Titrated with LFX. Each ITC experiment was repeated at least twice, representative thermogram and titration curve are shown.

Supplementary Figure S11

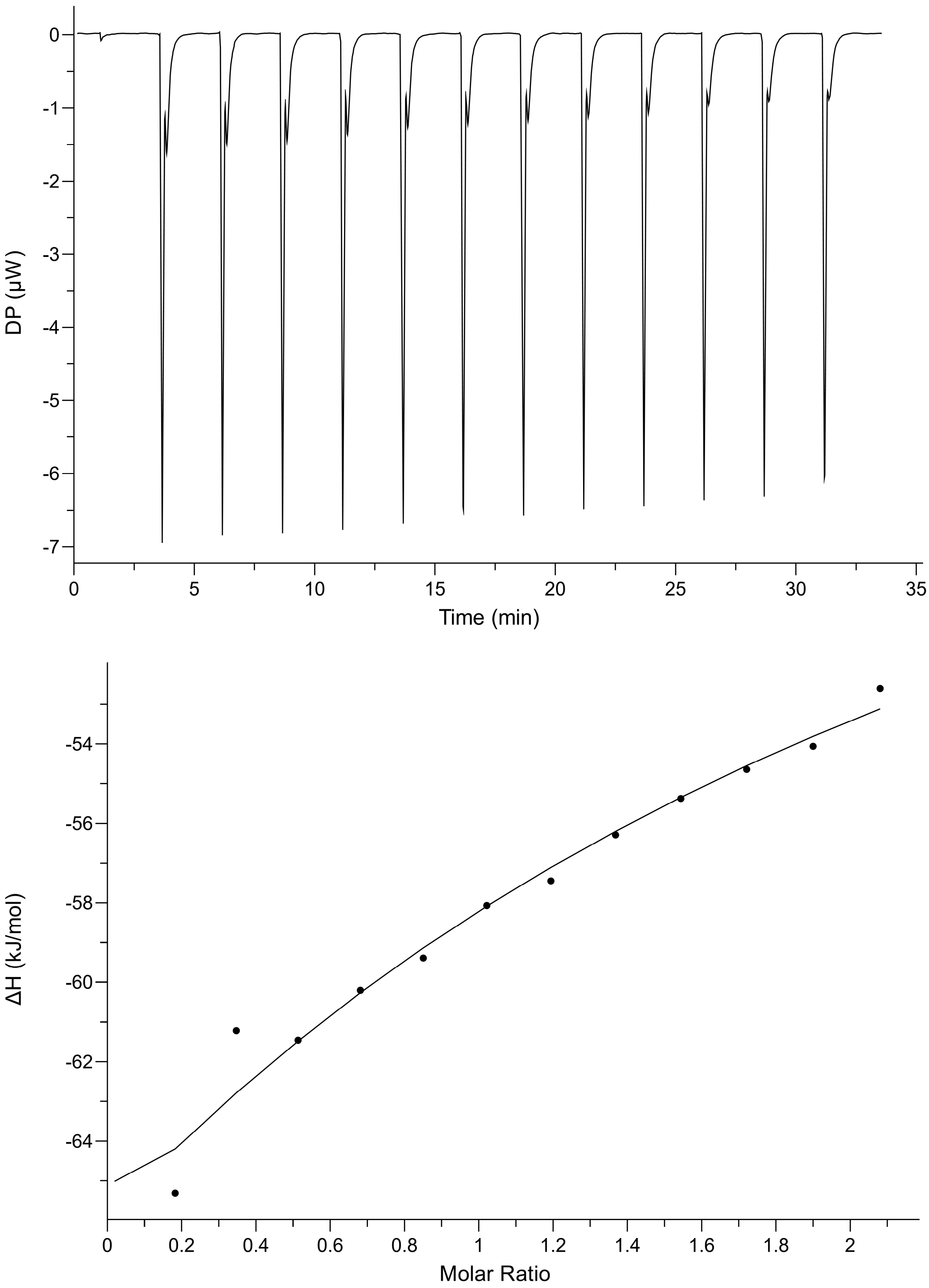

Figure S11. ITC thermogram and titration curve of the aptamer variant M6. Titrated with LFX. Each ITC experiment was repeated at least twice, representative thermogram and titration curve are shown.

Supplementary Figure S12

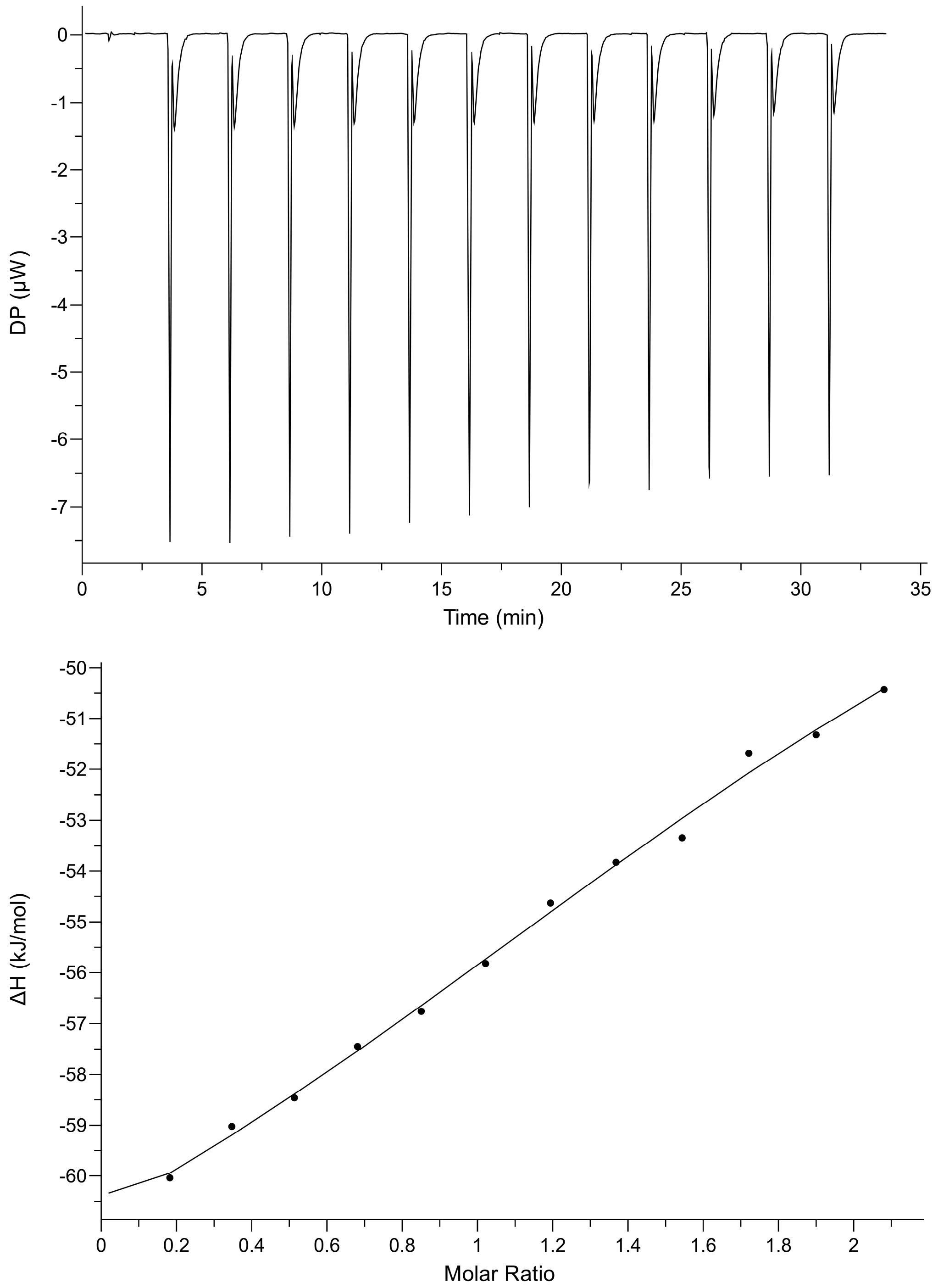

Figure S12. ITC thermogram and titration curve of the aptamer variant M7. Titrated with LFX. Each ITC experiment was repeated at least twice, representative thermogram and titration curve are shown.

Supplementary Figure S13

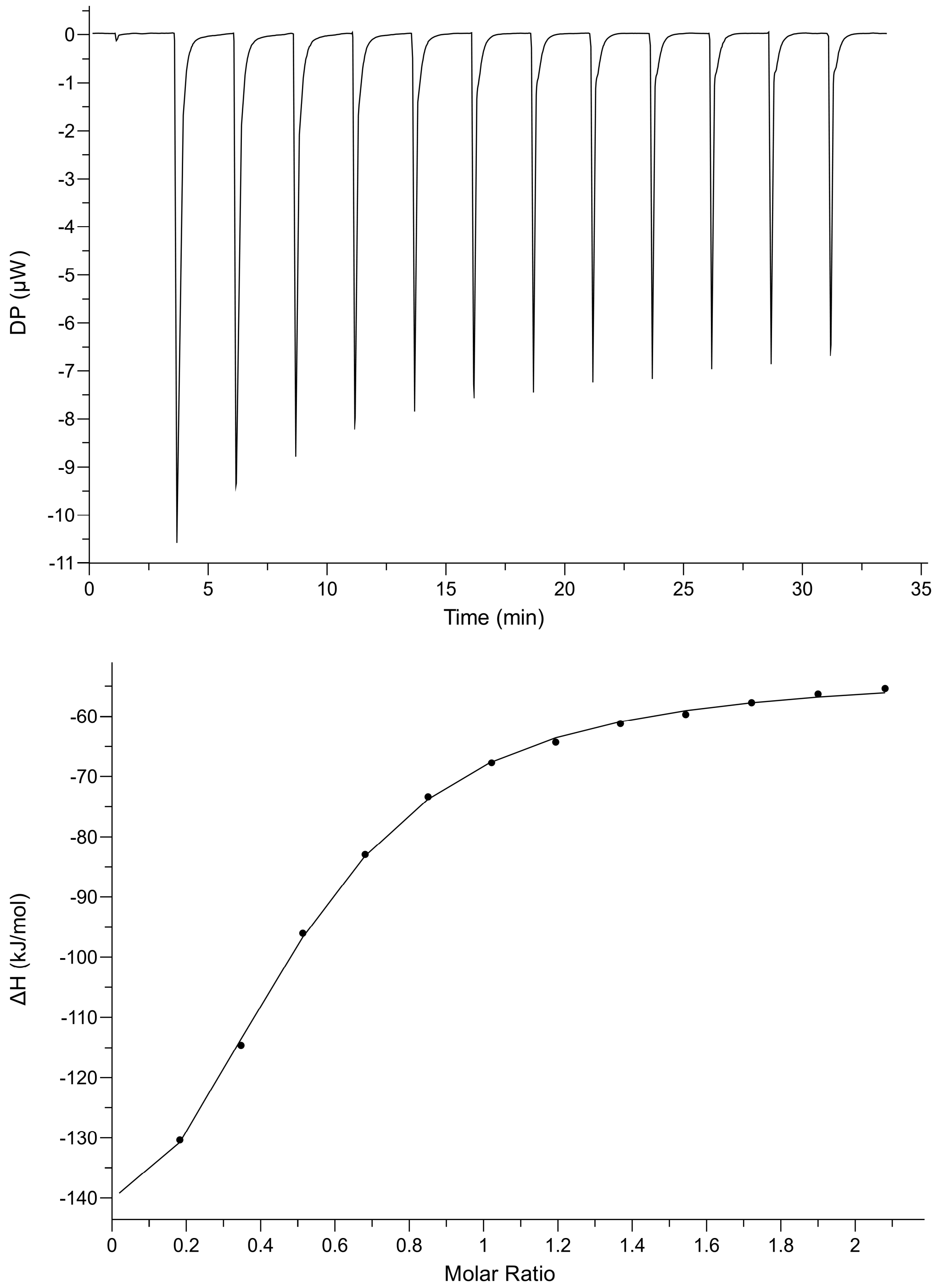

Figure S13. ITC thermogram and titration curve of the aptamer variant M8. Titrated with LFX. Each ITC experiment was repeated at least twice, representative thermogram and titration curve are shown.

Supplementary Figure S14

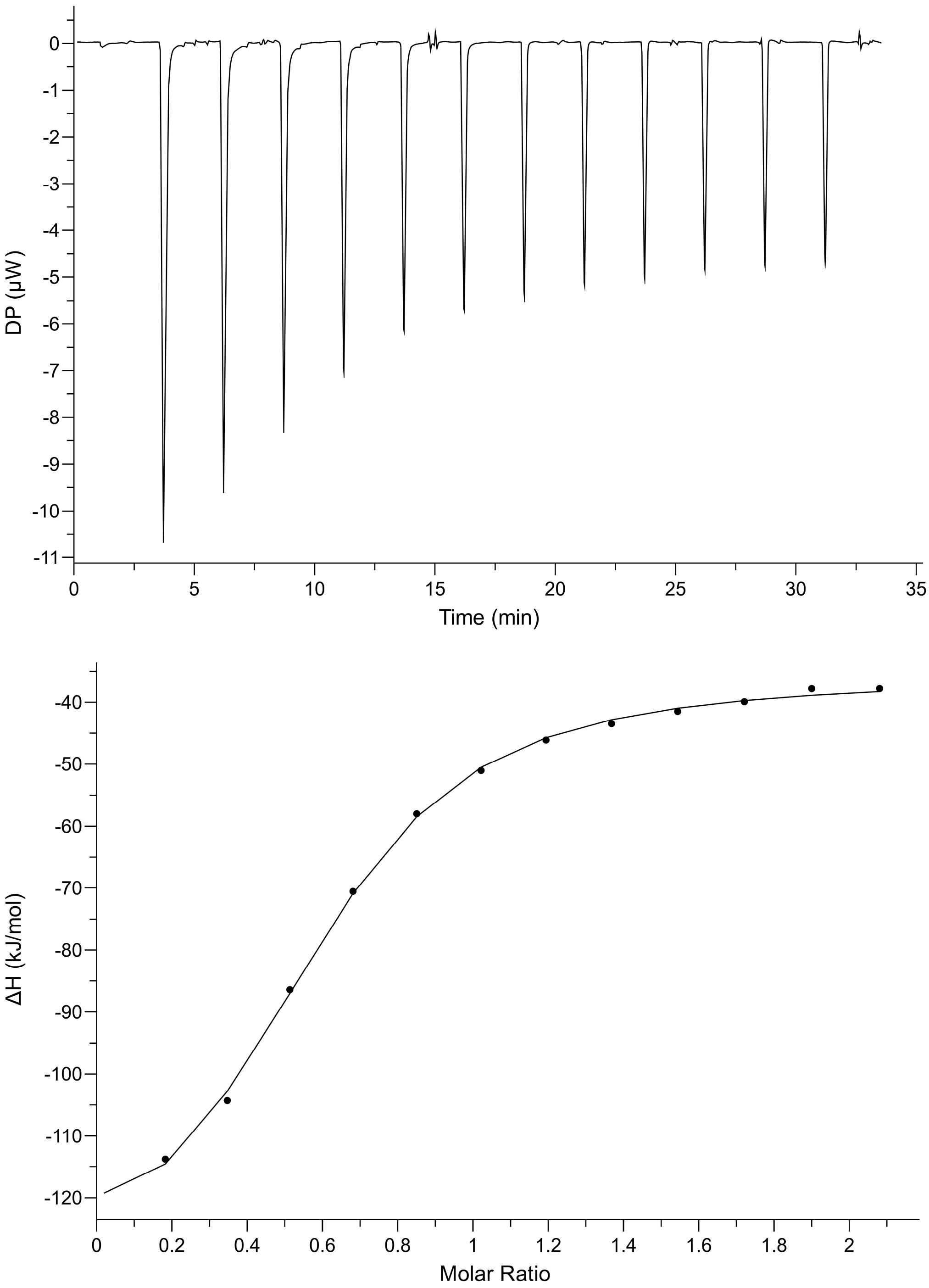

Figure S14. ITC thermogram and titration curve of the aptamer variant M9. Titrated with LFX. Each ITC experiment was repeated at least twice, representative thermogram and titration curve are shown.

Supplementary Figure S15

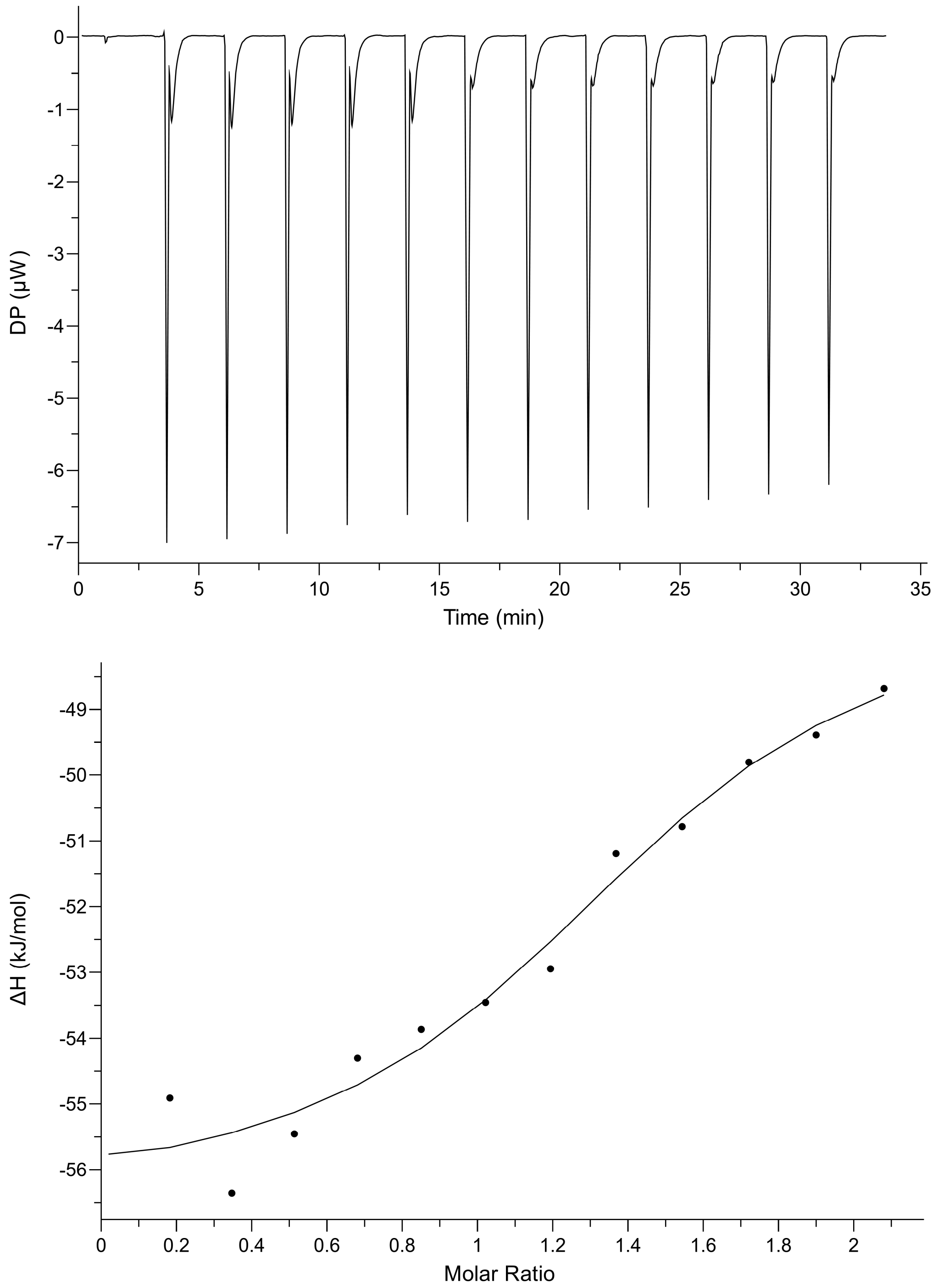

Figure S15. ITC thermogram and titration curve of the aptamer variant M10. Titrated with LFX. Each ITC experiment was repeated at least twice, representative thermogram and titration curve are shown.

Supplementary Figure S16

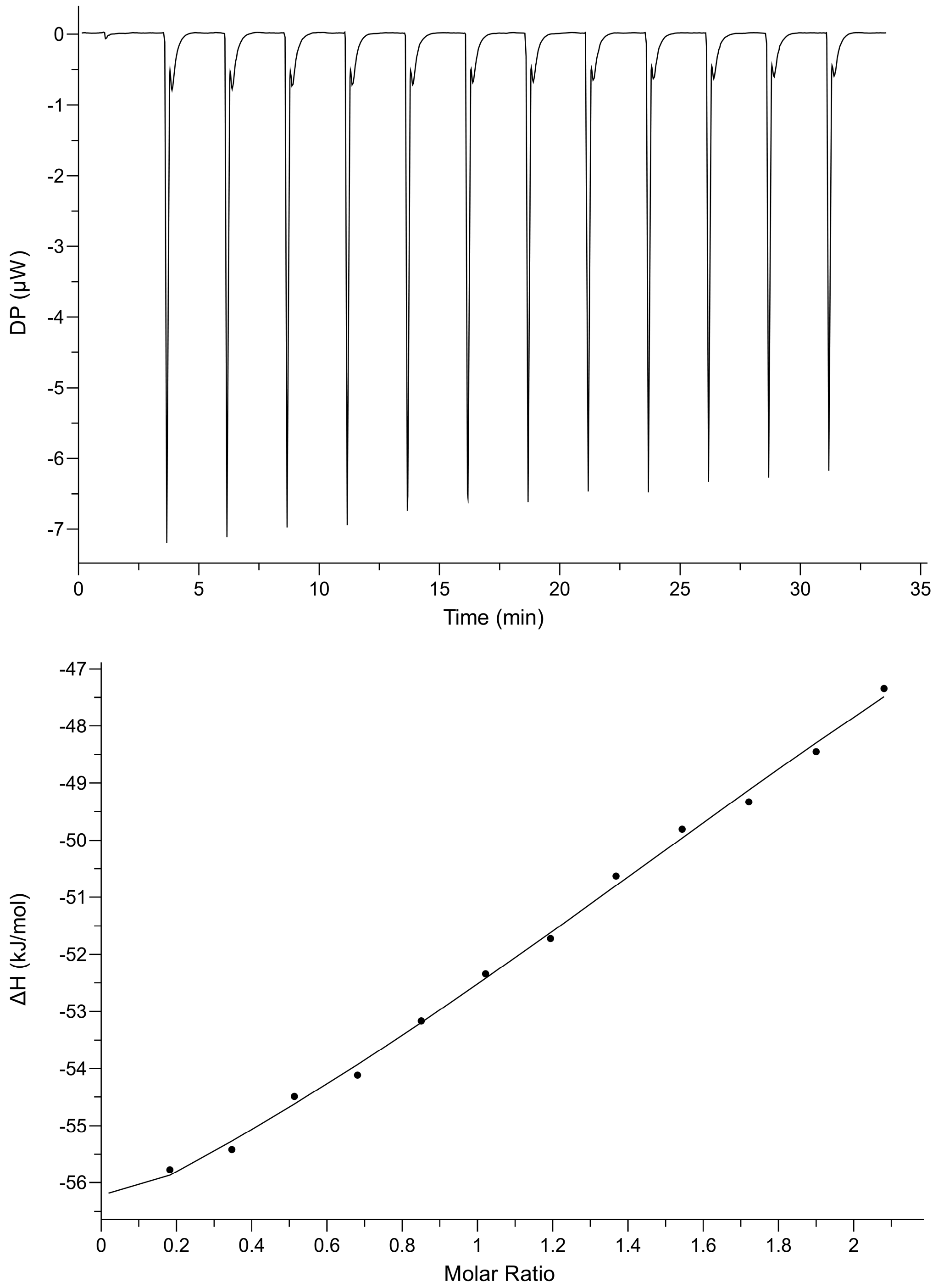

Figure S16. ITC thermogram and titration curve of the aptamer variant M11. Titrated with LFX. Each ITC experiment was repeated at least twice, representative thermogram and titration curve are shown.

Supplementary Figure S17

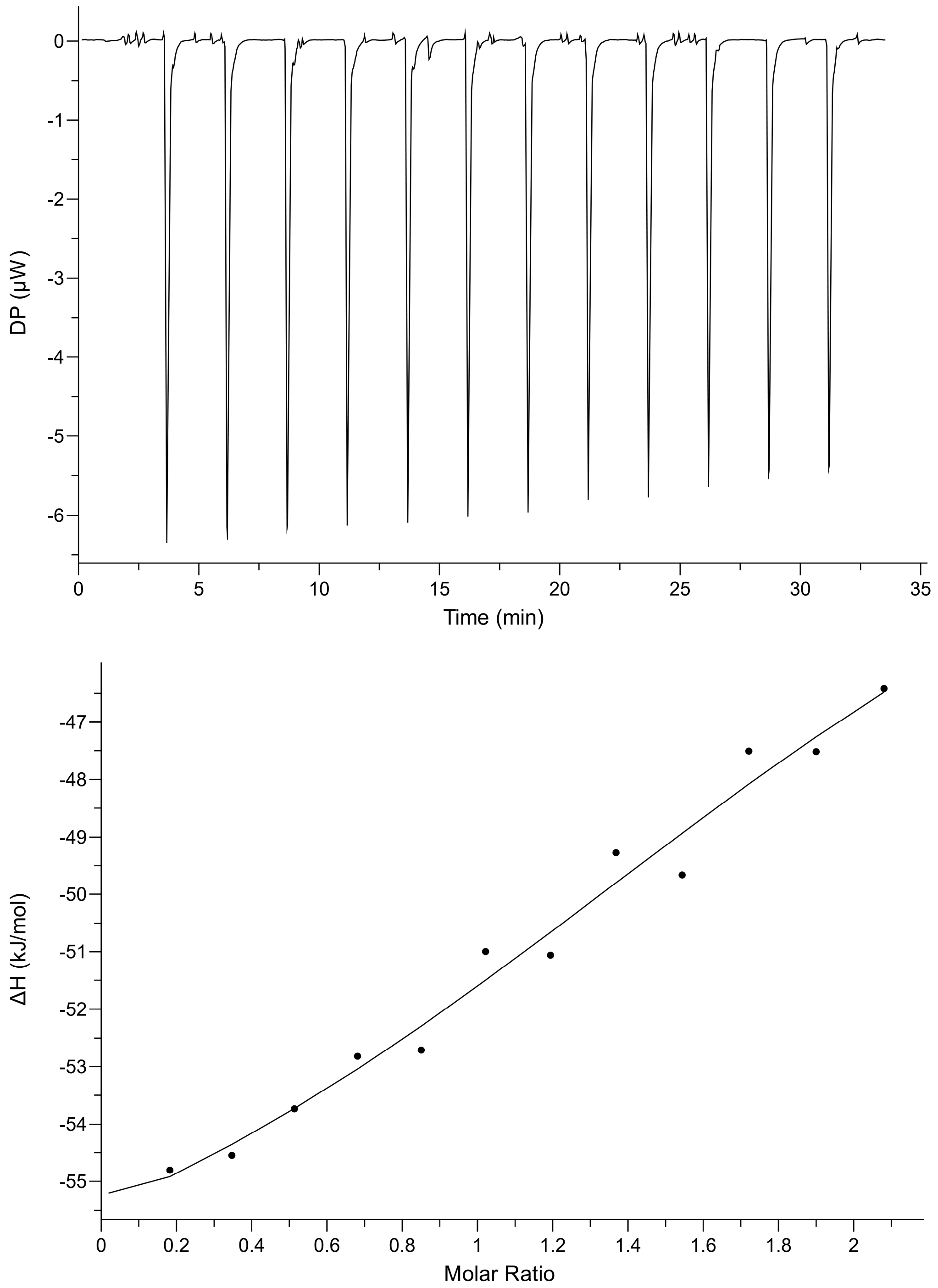

Figure S17. ITC thermogram and titration curve of the aptamer variant M12. Titrated with LFX. Each ITC experiment was repeated at least twice, representative thermogram and titration curve are shown.

Supplementary Figure S18

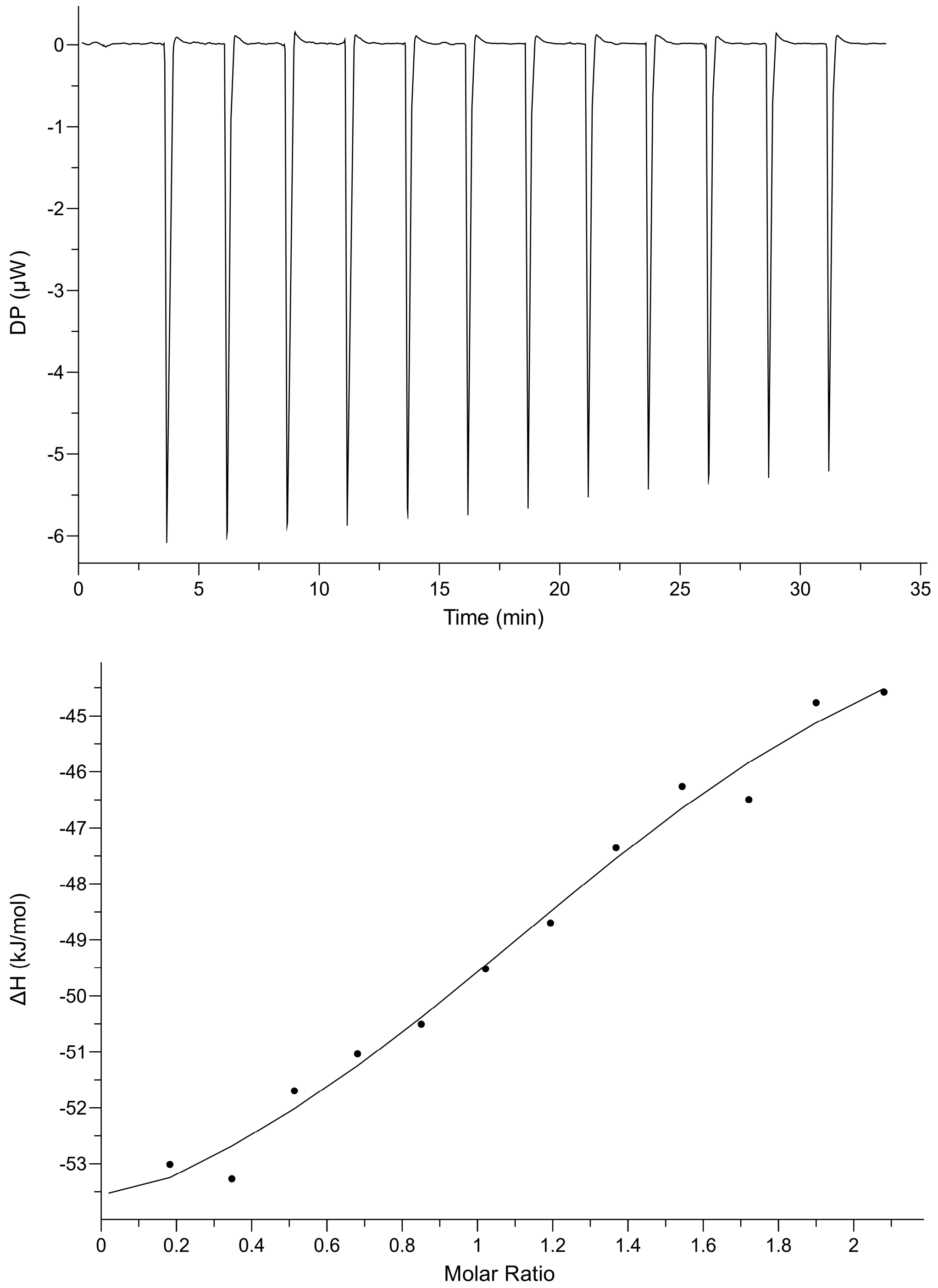

Figure S18. ITC thermogram and titration curve of the aptamer variant M13. Titrated with LFX. Each ITC experiment was repeated at least twice, representative thermogram and titration curve are shown.

Supplementary Figure S19

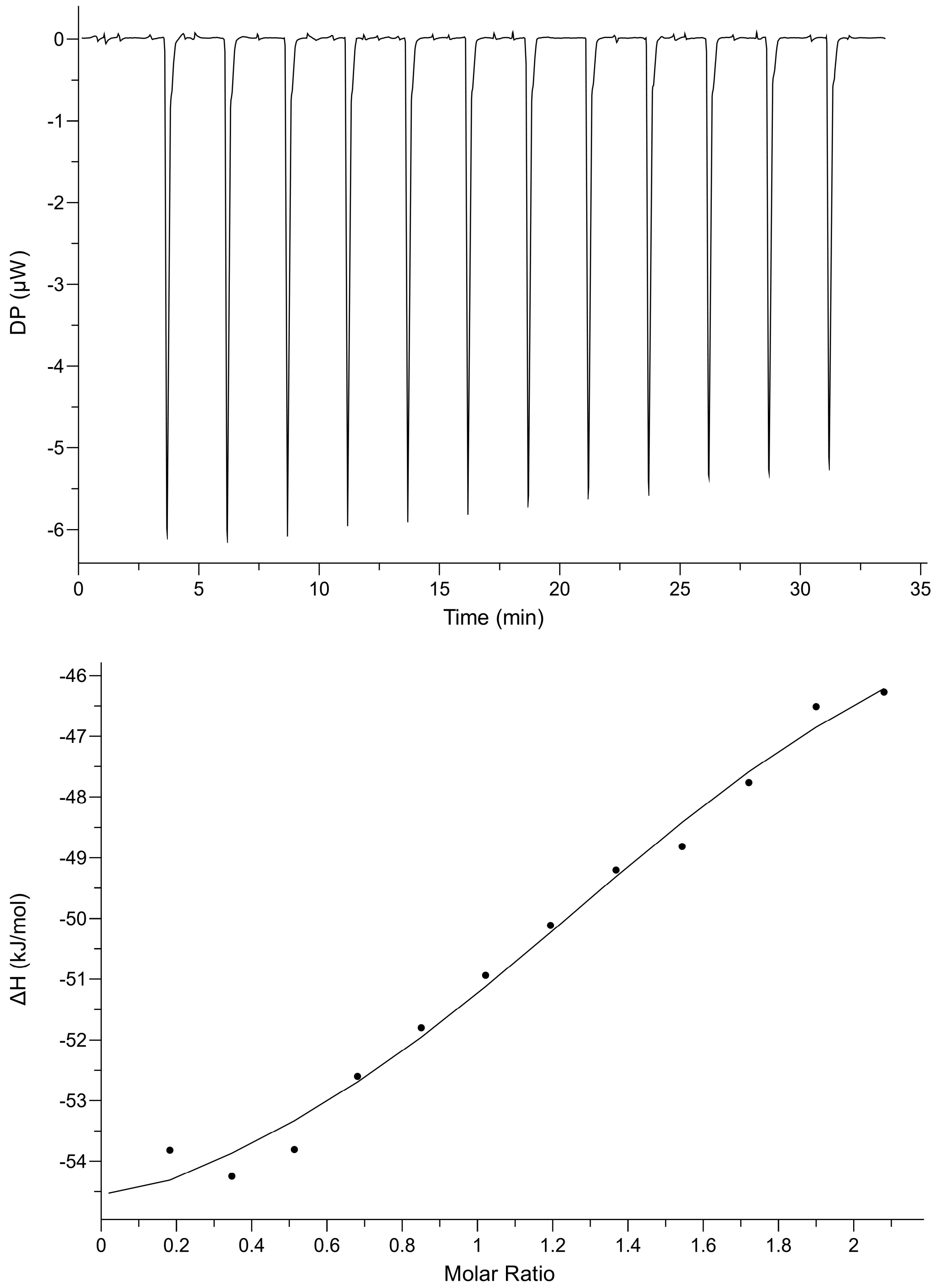

Figure S19. ITC thermogram and titration curve of the aptamer variant M14. Titrated with LFX. Each ITC experiment was repeated at least twice, representative thermogram and titration curve are shown.

Supplementary Table S1

Table S1. Results of 21 rounds and naive pool of merging paired-end reads using VSEARCH's mergepairs function.

Round	Pairs	Merged	Not merged	N	kmers	Mult. align.	diffs.	over-lap	Score	Exp. err.	too short	too long
0	130024	108355 (83.3%)	21669 (16.7%)	406	521	53	3	0	1676	467	3735	14808
1	153543	133916 (87.2%)	19627 (12.8%)	397	139	95	0	0	822	418	1957	15799
2	154376	136094 (88.2%)	18282 (11.8%)	428	151	27	2	0	833	514	1226	15101
3	148146	131259 (88.6%)	16887 (11.4%)	426	178	63	2	0	896	396	2003	12923
4	153819	135272 (87.9%)	18547 (12.1%)	425	152	21	2	0	999	701	1939	14308
5	159476	137771 (86.4%)	21705 (13.6%)	385	313	15	3	1	13903	525	2501	4059
6	129586	114085 (88.0%)	15501 (12.0%)	339	170	33	2	0	814	589	3950	9604
7	167780	146739 (87.5%)	21041 (12.5%)	498	185	26	2	0	875	639	6683	12133
8	179701	161985 (90.1%)	17716 (9.9%)	528	296	24	1	0	1097	780	10064	4926
9	169506	145588 (85.9%)	23918 (14.1%)	502	424	74	1	0	1174	548	16716	4479
10	169293	154579 (91.3%)	14714 (8.7%)	461	214	92	2	0	674	461	8358	4452
11	173891	164040 (94.3%)	9851 (5.7%)	435	249	203	1	0	788	627	3348	4200
12	181026	172716 (95.4%)	8310 (4.6%)	476	169	160	0	0	950	912	1941	3702
13	170565	162205 (95.1%)	8360 (4.9%)	454	263	283	1	0	887	631	1463	4378
14	76012	71102 (93.5%)	4910 (6.5%)	189	134	98	0	2	489	365	596	3037
15	117767	110733 (94.0%)	7034 (6.0%)	310	135	152	0	0	583	423	1157	4274
16	85773	71225 (83.0%)	14548 (17.0%)	8991	115	99	0	0	353	326	1329	3335
17	93418	87151 (93.3%)	6267 (6.7%)	264	149	206	1	0	525	333	600	4189
18	104205	97628 (93.7%)	6577 (6.3%)	231	226	263	2	0	533	285	756	4281
19	95134	89620 (94.2%)	5514 (5.8%)	241	139	282	0	0	471	340	668	3373
20	98617	92443 (93.7%)	6174 (6.3%)	275	215	245	0	2	610	491	954	3382
21	92650	86713 (93.6%)	5937 (6.4%)	218	162	335	1	0	571	332	639	3679

This table details the number of processed pairs (Pairs), merging efficiency (Merged, Not merged), and reasons for unsuccessful merges. Columns represent: 'N' for too many ambiguous nucleotides, 'kmers' for insufficient k-mer alignment, 'Mult. align.' for multiple alignment possibilities, 'diffs.' for high mismatches, 'overlap' for short overlap regions, 'Score' for low alignment scores or high score drops, 'Exp. err.' for high expected errors, 'too short' and 'too long' for merged fragments outside length thresholds.

Supplementary Table S2

Table S2. Post-Merging Sequence Filtering Results Over 21 Rounds and naive pool.

Round	Kept	Percentage
0	102223	0.94
1	129586	0.97
2	131965	0.97
3	122378	0.93
4	130598	0.97
5	132554	0.96
6	108446	0.95
7	140978	0.96
8	156546	0.97
9	137164	0.94
10	147984	0.96
11	158534	0.97
12	167994	0.97
13	155923	0.96
14	68925	0.97
15	107468	0.97
16	67918	0.95
17	83981	0.96
18	93685	0.96
19	87222	0.97
20	87184	0.94
21	84002	0.97

This table presents the count and percentage of sequences retained after filtering based on Levenshtein distance from a template, following VSEARCH post-merging and orientation processes.

Supplementary Table S3

Table S3. Number of Unique Sequence Counts Through Preprocessing Stages Over 21 Rounds Plus Naive Pool.

Round	Post-Merging	Post-Orientation	Post-Template Filtering
0	85615	85612	80783
1	105487	105480	102110
2	107338	107332	104067
3	103126	103120	96145
4	106086	106079	102399
5	108004	107963	103843
6	90176	89901	85443
7	110678	109565	105170
8	115568	113358	109112
9	81176	78414	72950
10	46403	43650	40708
11	23935	21620	19872
12	18328	16128	14721
13	17229	15213	13612
14	6801	5653	4906
15	9243	7674	6644
16	7588	6347	5254
17	8035	6658	5690
18	8324	6877	5789
19	7243	5954	5221
20	8351	6952	5643
21	7733	6387	5494

The number of unique sequences remaining after post-merging, post-orientation, and post-template filtering stages are shown.
